# Mistle: bringing spectral library predictions to metaproteomics with an efficient search index

**DOI:** 10.1093/bioinformatics/btad376

**Published:** 2023-06-09

**Authors:** Yannek Nowatzky, Philipp Benner, Knut Reinert, Thilo Muth

**Affiliations:** Section S.3 eScience, Federal Institute for Materials Research and Testing (BAM), Berlin 12205, Germany; Section S.3 eScience, Federal Institute for Materials Research and Testing (BAM), Berlin 12205, Germany; Department of Mathematics and Computer Science, FU Berlin, Berlin 14195, Germany; Department of Computational Molecular Biology, Max Planck Institute for Molecular Genetics, Berlin 14195, Germany; Section S.3 eScience, Federal Institute for Materials Research and Testing (BAM), Berlin 12205, Germany

## Abstract

**Motivation:**

Deep learning has moved to the forefront of tandem mass spectrometry-driven proteomics and authentic prediction for peptide fragmentation is more feasible than ever. Still, at this point spectral prediction is mainly used to validate database search results or for confined search spaces. Fully predicted spectral libraries have not yet been efficiently adapted to large search space problems that often occur in metaproteomics or proteogenomics.

**Results:**

In this study, we showcase a workflow that uses Prosit for spectral library predictions on two common metaproteomes and implement an indexing and search algorithm, Mistle, to efficiently identify experimental mass spectra within the library. Hence, the workflow emulates a classic protein sequence database search with protein digestion but builds a searchable index from spectral predictions as an in-between step. We compare Mistle to popular search engines, both on a spectral and database search level, and provide evidence that this approach is more accurate than a database search using MSFragger. Mistle outperforms other spectral library search engines in terms of run time and proves to be extremely memory efficient with a 4- to 22-fold decrease in RAM usage. This makes Mistle universally applicable to large search spaces, e.g. covering comprehensive sequence databases of diverse microbiomes.

**Availability and implementation:**

Mistle is freely available on GitHub at https://github.com/BAMeScience/Mistle.

## 1 Introduction

Metaproteomics is a key technology for characterizing proteins in complex microbial samples at a given time point (Wilmes and Bond 2004) and can provide pivotal information about taxon-specific functional activity, as well as signaling and metabolic pathways within the microbial community (Hettich *et al.* 2013, Tanca *et al.* 2017). This enables studying health and disease cases of host species, and ecological dynamics in all kinds of biological systems and microbiomes (Hettich *et al.* 2013, Scholz *et al.* 2015, Callieri *et al.* 2018). Inherent to the proteomic investigation of microbial communities is a large search space, because many species, often previously unknown ones, are present in a typical microbiome sample and need to be queried (Schiebenhoefer *et al.* 2019).

Peptide identification lies at the heart of high-throughput proteomics workflows, where the collected sample of proteins is usually subjected to enzymatic digestion and liquid chromatography (LC) coupled with tandem mass spectrometry (MS/MS) (Coon *et al.* 2005, Hettich *et al.* 2013). Various search algorithms have been designed to identify the underlying peptides from the MS/MS spectra in a protein sequence database, assigning quality scores for so-called peptide spectrum matches (PSMs) (Verheggen *et al.* 2020). However, distinguishing true identifications from false positive hits becomes increasingly hard with increasing size of the protein database that is used as a reference for peptide identification (Verbruggen *et al.* 2021). A greater number of potential matches make it statistically more likely that a random false match receives a higher score than the true match (Nesvizhskii 2010, Verbruggen *et al.* 2021). When filtering for false positives, an increase in database size may lead to a reduced number of peptides identified. Even multi-stage search strategies, which aim to reduce the search space by tailoring the database through multiple search steps, may at the same time invoke more false discoveries (Muth *et al.* 2015, Verheggen *et al.* 2020). Evidently, there is a demand to overcome this inherent weakness of database search when facing large search spaces.

Due to the diversity of species and genera found in microbial communities, metaproteomic studies are especially resource-straining for common database search engines, such as MSFragger (Kong *et al.* 2017), which compare MS/MS scans to theoretical fragment ions of peptides in the sequence database. This is noticeable not just in terms of reduced sensitivity in peptide identification, but also in increased run time, and even more prominently in memory requirements by the algorithms, due to the large candidate spaces.

Machine learning approaches have been implemented to enhance peptide identification, e.g. by post-processing the database search results (Verbruggen *et al.* 2021). A particularly prominent example of this is Percolator (Käll *et al.* 2007), which uses semi-supervised learning with support vector machines. More recently, deep learning models such as pDeep (Zhou *et al.* 2017) and Prosit (Gessulat *et al.* 2019) predict complete mass spectra including fragment intensities and retention time from peptide sequences, and thus offer a method to rescore database search results based on these predicted spectral features, coupled with Percolator.

Consequently, Prosit also provides the means to predict spectral libraries for entire proteomes, which can then be queried. As of now, metaproteomics has not yet had a chance to fully make use of such comprehensive prediction workflows covering the proteomes of many species, as this leads to massive spectral libraries. Current spectral library search software, such as SpectraST (Lam *et al.* 2007), is not equipped to meet run time and memory constraints imposed by such large MS/MS databases, covering >10 000 000 peptide spectrum predictions. At the same time, metaproteomics could benefit from more precise search algorithms, as the large search space has been shown to reduce sensitivity and exacerbates challenges with false discovery estimation in large metaproteomic settings (Muth *et al.* 2015). In fact, Gessulat *et al.* (2019) mention the use case of Prosit for metaproteomics, and manage to improve database search results by rescoring the top Andromeda hits (Cox *et al.* 2011) using the spectral predictions. In 2021, Verbruggen *et al.* presented a solution for large search spaces in proteogenomics, for ribosomal profiling, by using predicted spectral features to enhance identification rate and stringency in PSMs. However, to this day there is no efficient workflow to apply complete spectral library predictions to metaproteomics and efficiently search such substantial amounts of MS/MS data.

We propose such a workflow using a predicted library and directly search for the best matching peptide using spectral similarity measures. First, we digest the complete metaproteome sequence database (*in silico*) with EncyclopeDIA (Searle *et al.* 2020) and then use Prosit to predict MS/MS spectra for every peptide and charge configuration that is reasonably likely to occur in an MS/MS run. Finally, we use our novel **M**etaproteomic **i**ndex and **s**pec**t**ral **l**ibrary search **e**ngine, short Mistle, to query the spectral library.

Mistle creates a small, searchable index and is extremely run time and memory efficient. We achieve this by adapting the fragment index of MSFragger to spectral intensity matching. Additionally, we introduce an advanced index partitioning and query scheduling method to the algorithm and add hardware optimization, such as SIMD intrinsics in combination with multithreading, to greatly reduce memory footprint and run time.

This workflow virtually turns the sequence database search problem into a spectral library search problem. We benchmark the algorithmic performance of Mistle with state-of-the-art methods and examine the potential of our workflow to qualitatively and quantitatively improve metaproteomic studies on the peptide identification level, based on two sample metaproteomes, the lab-assembled nine-organism microbial mixture (9MM) by Tanca *et al.* (2013) and the extended simplified human intestinal microbiota (SIHUMIx) sample by Krause *et al.* (2020).

## 2 Materials and methods

Mistle is inspired by the fragment index data structure introduced by Kong *et al.* (2017) and employed in MSFragger. Instead of iteratively matching experimental spectra with every theoretical spectrum calculated from candidate peptide sequences, MSFragger constructs an index that stores theoretical fragment ions in a rapidly searchable format, enabling fast and simultaneous peak matching for peptide candidates. We adapt the core idea to the spectral search problem, where instead of a protein sequence database a predicted MS/MS library is queried.

While the main idea remains the same, i.e. searching fragments in the fragment index and updating scores of their parents, which are now MS/MS spectra rather than peptides, there are significant additional challenges to overcome. For one, peak intensities must be considered and stored in the fragment index. This immediately makes the fragment index larger, which poses tangible memory problems for metaproteomics libraries and slows down processing because intensities must be multiplied. Simply counting and summing up intensities, as it is the case for MSFragger, is no longer sufficient. Also, the index needs to be constructed from a spectral library, which is data-intensive to a point where it is infeasible to hold all data in random access memory (RAM). Thus, information required to construct the data structures needs to be carefully and continuously conveyed throughout the reading process to produce the final index.

Here, we introduce algorithmic solutions to all these hurdles and propose optimizations to counteract increased run time arising from the additional multiplication-operations when matching peaks. Mistle is implemented from scratch in C++ 20 and features single instruction multiple data (SIMD) extensions.

### 2.1 Data structures

#### 2.1.1 Precursor index

Similarly to MSFragger, we require an auxiliary data structure, referencing all library targets, i.e. peptides linked with their predicted MS/MS spectra. We call it precursor index, as entries are searchable by the precursor peak. It is equivalent to the peptide index described by Kong *et al.* (2017).

Specifically, the precursor index stores a unique identifier (ID, 32-bit unsigned integer) for every mass spectrum in the library, which each corresponds to exactly one peptide. The IDs are ordered by the precursor’s charge and mass-to-charge ratio (*m*/*z*). This serves as a reference for the fragment index. Additionally, a mapping must be provided from the ID to the rank of the spectrum in the precursor index. This is the inverse of the sorted precursor IDs. We implement this as an additional lookup vector, precomputed at index construction by a linear scan over the ranked ID vector.

#### 2.1.2 Fragment index

In the fragment index, all fragment ions *f*, i.e. peaks, of library MS/MS spectra are stored in form of triplets, of the ion mass (*m*/*z* value, $mz_{f}$), peak intensity ($I_{f}$) and the unique ID of the underlying parent spectrum: $f=(mz_{f},I_{f},{\text{ID}}_{\text{parent}}(f))$. The latter provides a reference from fragment to the matched peptide/spectrum and facilitates an efficient search of peaks from matching candidate spectra.

**Figure 1.**
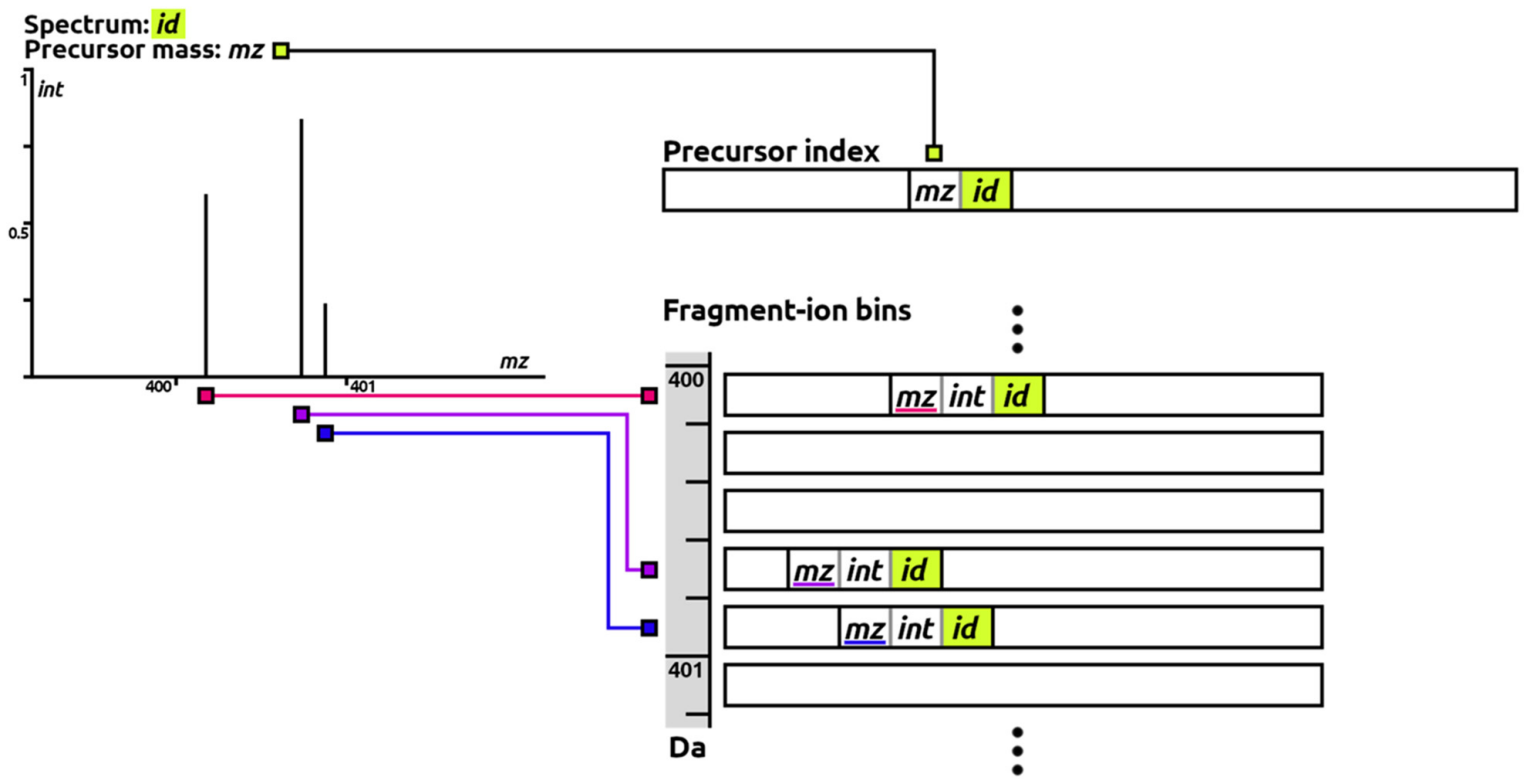
Precursor and fragment index data structures at construction. For an exemplary library spectrum (top left), it is shown how the precursor is included in precursor index sorted by mass (*mz*) and referenced by a unique identifier (*id*). All peaks are integrated in the fragment-ion bins corresponding to their ion mass and encoded as triplets [*m*/*z* value (*mz*), intensity (*int*) and parent ID (*id*)]. Their order inside each bin is determined by the rank of the parent in the precursor index.

For that to be possible, the fragments (triplets) are placed into bins based on their ion mass, given an adjustable bin width *B*. Inside each bin, fragments are sorted according to the parent rank in the precursor index, accessed via the parent ID. This way, for a certain mass range [M,M+B), every peak with $mz_{f}\geq M$ and $mz_{f}<M+B$ of the entire spectral library is stored in the corresponding fragment bin. The order of those peaks allows for a division based on parent mass. The structure of the precursor and fragment index are illustrated in Fig. 1.

#### 2.1.3 Partitioning

As the union of precursor index and fragment index holds about as much information as the entire spectral library, the required index space grows linearly with the database size and needs to fit into main memory for efficient access. To make a search feasible for large reference libraries, we propose partitioning the main part of the index, i.e. fragment index, into several smaller sub-indices or partitions. Such a technique has been shown to be quite effective for other bioinformatic problems, showcased for instance by the DREAM index framework (Dadi *et al.* 2018). Ideally, each query spectrum only needs to be searched in one or a small number of partitions, which combined retain the original index data structure.

We achieve this by creating separate fragment bins for each partition, which we tie to non-overlapping precursor *m*/*z* intervals. A fragment triplet is stored in its corresponding fragment bin, for the partition only, where the parent’s precursor peak falls into the *m*/*z* interval. Each partition has the full number of fragment bins, and acts as an individual fragment index. This way, a query spectrum only needs to be searched in a partition matching its precursor mass, within a given *m*/*z* tolerance. Also, within each partition the search algorithm can be performed identically. Merely the number of library spectra included is reduced for each partition. This not only reduces physical space that needs to fit into the main memory at a time, but also the search space for a given query within the partition. Fewer comparisons are needed during the binary search, explained in Section 2.2.

#### 2.1.4 Continuous index construction algorithm

As mentioned before, the input library might be arbitrarily large and in no particular order. When reading the data, the precursor index, which is necessary to order all fragments, is unknown, up until the very end. A practical, memory efficient approach is to create preliminary (unsorted) index partitions on the disk when reading the library and to update the partitions once all relevant information has been obtained. A detailed description of the process can be found in the Supplementary Text.

### 2.2 Search algorithm

Partitioning the fragment index creates an initial overhead when searching experimental spectra, because spectral queries need to be scheduled to relevant partitions and merged afterwards. This is performed by assigning each experimental query spectrum a unique identifier and constructing a list of query IDs for each partition to address, based on the precursor *m*/*z* and mass tolerance. Then, each partition with at least one query scheduled is loaded into main memory, and spectral matching is performed.

**Figure 2.**
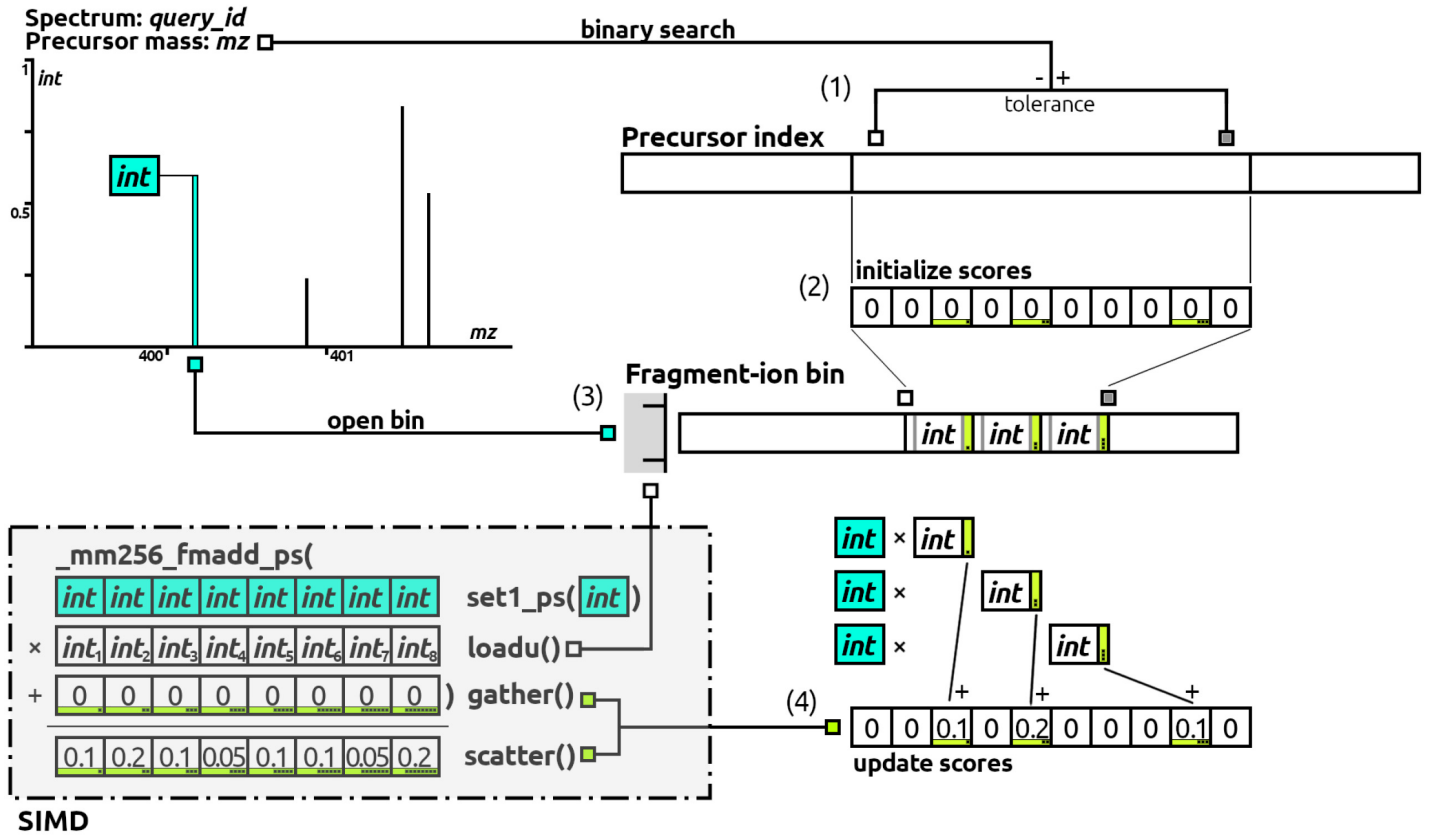
Illustration of the search process: matching an exemplary query spectrum (top left) to all indexed library spectra. First, the binary search step (1) is shown on the precursor index, where the lower and upper bound of candidate spectra is determined and an empty scoring vector is initialized (2). Thereafter, the peak-by-peak matching is shown for the fragment index, highlighted for the first query peak (turquoise) and the corresponding fragment bin. (3) Here, a binary search is performed to determine relevant matches with a parent rank within the lower and upper bound. (4) Lastly, fragment intensity entries are multiplied to the peak intensity and added to the respective parent scores. SIMD intrinsics may replace step 4, as shown on the bottom left, e.g. computing eight intensity products and adding them to the dot products in a single CPU operation.

Initially, matches are ranked by the spectral dot product of normalized intensities, as described in Eq. (2) by Lam *et al.* (2007). The similarity scoring function is refined later on. Raw intensity values are square rooted before normalization to de-emphasize dominant peaks. By definition, a peak only contributes to the dot product, if a matching peak from the other spectrum exists in the same *m*/*z* bin. Conversely, the dot product needs to be updated only for those reference spectra that have a fragment entry in the corresponding fragment bin. A binary search going through that fragment bin quickly narrows down the calculation to exactly those candidates that lie within the precursor *m*/*z* range. Essentially, we leverage the data structures from Section 2.1 to perform a fast search, reminiscent of MSFragger’s fragment index search, but compute the spectral dot product in the process, as is illustrated in Fig. 2. The overt novelty lies within matching fragments by their intensities, in addition to the *m*/*z* dimension, when iterating a fragment bin. The intensity product ($I_{p}I_{f}$) of query peak $p=(mz_{p},I_{p})$ and fragment *f* is added to the parent score, which is accessed by the parent identifier ${\text{ID}}_{\text{parent}}(f)$. As this is computationally costly, we speed up the arithmetic operations using SIMD extensions. The fused multiply-add operation (in C++: _mm256_fmadd_ps), available for the Advanced Vector Extensions AVX2 and AVX512 architectures, allows parallel multiplication and addition of 8–16 floating-point numbers in a single CPU instruction. A schematic workflow with the use of SIMD for a 256-bit register is depicted in Fig. 2, bottom left. Moreover, the search loop is parallelized matching each query spectrum on a separate thread.

After ranking all candidate spectra with the fragment index, we reevaluate the top hits, tracking all kinds of statistics. A refined *bias-adjusted similarity* measurement, which resolves *m*/*z* bins by modeling peak intensity spread with a Gaussian bell curve, determines the X highest scoring library spectra (see Section 2.2.1 for a detailed description). X, the number of output PSMs per query spectrum (X > 0), is a parameter defined by the user. A step-by-step walk-through of the search loop including the implementation of SIMD with intrinsic C++ functions are discussed in the Supplementary Text.

Once all scheduled queries are performed, the resulting PSMs from all the partitions are concatenated and sorted by query ID. Matches assigned to the same experimental spectrum cluster together, and again only the top X ranked matches are retained, if a query was carried out in multiple partitions.

#### 2.2.1 Spectral similarity scoring

At its core, Mistle provides high-performance spectral matching based on the spectral dot product of binned peaks [Eq. 2 by Lam *et al.* (2007)]. Precursor *m*/*z* tolerance and bin width are parameters adjustable by the user. However, for any search engine to distinguish true from false matches, the choice and design of scoring functions and spectral processing steps are essential. Currently, the only pre-processing operations applied to the spectra are square root transformation and normalization of peak intensities. A top-k in window-w noise reduction approach is implemented for optional use. Top ranked matches for each query are rescored providing a multitude of scores including shared peak counts, a log-hyperscore, an *f*-value equivalent as seen in SpectraST, and various Mistle specific scores. Differences and refinements made to spectral similarity scoring are described below.

Peak intensity spread over multiple bins (as is implemented in SpectraST) to account for slight shifts in peak *m*/*z* is inefficient with the proposed fragment index. Instead, we resolve the initial binning when rescoring matches. Our formula models peak intensity spread with a Gaussian bell curve put over the peaks and penalizes mass shifts as intensity decay along the curve. We define a similarity between a query spectrum *Q* and reference spectrum *R*, analogous to the dot product of binned intensities: and where *k* iterates over all reference peaks $(mz_{k},I_{k})\in R$ and *l* over all query peaks $(mz_{l},I_{l})\in Q$. ϕ(x,μ) factors in the distance of *mz*-values given the standard deviation σ between peaks. We set σ, which models the fragment tolerance, equal to bin size. An analogous formula to the dot bias as provided by SpectraST (Lam *et al.* 2007) can be established:


(1) $$\;\mathrm{similarity}(Q,R)=\sum_{k}\max_{l}\;I_{l}I_{k}\phi (mz_{l},mz_{k}),$$

(2) $$\phi (x,\mu )=\text{exp}\;\left(-\frac{1}{2}{\left(\frac{x-\mu }{\sigma }\right)}^{2}\right),$$

(3) $$\;\mathrm{bias}(Q,R)=\frac{1}{\mathrm{similarity}(Q,R)}\sqrt{\sum_{k}(\underset{l}{\max\;}I_{l}I_{k}\phi (mz_{l},mz_{k}){)}^{2}}.$$

For the purpose of this study, we employ a *bias-adjusted similarity* measurement as: similarity⋅(1−bias).

Note that as Prosit only predicts *b* and *y* ion intensities, it might be ill-advised to score similarity based on all peaks. A correct match might achieve an inferior score, when additional ion-type peaks are present, which are not matched by the prediction. Therefore, we introduce a second type of similarity, which only considers similarity of matched peaks, which we call *reflection score*. All unmatched peaks from the experimental spectrum are considered noise and do not influence this score. Bias and *bias-adjusted similarity* are formulated equivalently on matching ions only. The final scoring function for the evaluation of target decoy competition is the average between *bias-adjusted similarity* and the *reflection score* version of the same formula. Remember that the *reflection score* alone might not provide a perfect distinction either, since it can disregard large portions of the experimental MS/MS spectrum. A false peptide might achieve a high score by matching well to small peaks annotated as *b* and *y* ions yet leaving most of the peak intensity unaccounted for. Thus, we opt for the average out of both spectral similarity measurements.

### 2.3 Data preparation

The datasets in this study were derived from sources in the public domain. Identifiers and links to the data are provided below and in the Supplementary Text. We evaluate the performance of Mistle on the NIST human (*Homo sapiens*) consensus library (downloaded from doi.org/10.18434/T4ZK5S; Instrument: Ion Trap; Build date: 05-29-2014) and two common mock communities, 9MM (Tanca *et al.* 2013) and SIHUMIx (Krause *et al.* 2020). For the latter, we follow the recently published CAMPI study (Van Den Bossche *et al.* 2021), such that the evaluation is on par with the current metaproteomic benchmarking standard.

Protein sequence databases are re-utilized from Tanca *et al.* (2014) and the CAMPI study. Four original search files for 9MM and two large search files from CAMPI are selected for the comparison. Additionally, a yeast (*Saccharomyces cerevisiae*) consensus library by NIST (downloaded from doi.org/10.18434/T4ZK5S; Instrument: Ion Trap; Build date: 04-06-2012) serves as an experimental ground truth dataset. A summary of the two microbiomes and the data is found in the Supplementary Text.

The human spectral library is queried with 18 search files from the human HEK293 cell line [Roos *et al.* (2016); PRIDE ID: PXD001197] aligning our study with the spectral benchmarking by Wang *et al.* (2020) that already compared msSLASH and SpectraST.

#### 2.3.1 Spectral library construction

The human spectral library is set up with SpectraST (in .mgf format) with the corresponding decoy library generated using the decoy precursor swap method by Cheng *et al.* (2013). In total, the library consists of 339 970 target precursors and 339 942 decoy precursors.

Spectral libraries covering both microbiomes are predicted with the following workflow: The protein sequence databases are digested using EncyclopeDIA (Searle *et al.* 2020) with a mass range of 400–1500 Da, charge states 2–4, and up to two missed cleavages. The normalized collision energy (NCE) is left at default value 33. Then, a locally installed version of Prosit, downloaded from https://github.com/kusterlab/prosit in 2019, is used to predict MS/MS spectra for all peptides and charge conformations in the peptide list. This way, the only modification considered is Cysteine Carbamidomethylation, which is fixed. A decoy library, when necessary, is created using DecoyPyrat (Wright and Choudhary 2016) with minimum peptide length 7, and the downstream procedure is executed identically. Note that the SIHUMIx database already contains decoy sequences. Here, the database is split into two separate sets instead, before digestion and prediction. For 9MM, the contaminants database cRAP is appended downloaded from the GPM FTP site http://ftp.thegpm.org/fasta/cRAP/ in December 2021. Additionally, the human proteome, downloaded from Uniprot Proteomes (Proteome ID: UP000005640), is added as an entrapment database to the target sequences. Again, we produce corresponding decoys with the method described above and digest and predict the spectral libraries accordingly.

All of this results in 9 995 224 human peptide spectra predicted by Prosit, 10 630 095 spectra from 9MM and 7 806 271 spectra from SIHUMIx species (51.8 GB in .msp format in total). The Supplementary Material contains additional statistics regarding all datasets.

#### 2.3.2 Search setup

The *mistle-build* (v0.1.1) indexing algorithm is applied to the data creating a searchable index with 64 search partitions in condensed binary format for the target and decoy library. The four experimental 9MM files are then searched using the *mistle-search* (v0.1.1) program with 10 ppm precursor tolerance and 0.2 Da fragment tolerance (bin size), as suggested by Tanca *et al.* (2013). The yeast consensus spectra were searched at 10 ppm precursor tolerance. Here, we relaxed the fragment tolerance to 0.5 Da, as the machine accuracy is unknown and a higher fragment tolerance was found to perform better. The two experimental files from the CAMPI study are searched in the SIHUMIx library with 10 ppm precursor tolerance and 0.02 Da fragment tolerance as was done by Van Den Bossche *et al.* (2021).

We conduct the exact same searches with SpectraST and msSLASH (Wang *et al.* 2020) on the target and decoy libraries, and with MSFragger given the original protein sequence databases. MSFragger 3.4 was used via the FragPipe pipeline. SpectraST version 5.0 was installed together with the TPP v6.0.0 software (Deutsch *et al.* 2015) and msSLASH was downloaded from GitHub (https://github.com/COL-IU/msSLASH). Precursor and fragment tolerances are set as described above, peptide mass ranges are defined accordingly, and modifications are set to carbamidomethylated Cysteine only, to ensure a fair comparison. Aside from that, all tools run with default parameters. All pre-processing steps and mass calibrations are allowed. Since SpectraST and msSLASH accept precursor tolerance only in absolute values, we set it to 0.015, so that it considers all candidates for the largest peptides (10 ppm of 1500 Da).

#### 2.3.3 Quality control

False discovery rate (FDR) estimation using target decoy competition is put in place as primary technique to ensure high quality of identification. For separate target and decoy searches (Mistle and SpectraST) the results are first merged, retrieving only the top scoring hit, either from the target or the decoy library. The FDR is then estimated from the number of target peptides $N_{\text{target}}$ emitted at any scoring threshold *t* and corresponding the number of decoys $N_{\text{decoy}}$, as a measure of false discoveries $N_{\text{false}}$ among them: $N_{\text{false}}$ is directly measurable only if the correct peptides for the MS/MS spectra are specified.


(4) $$\text{FDR}(t)=\frac{N_{\text{false}}(t)}{N_{\text{target}}(t)}\approx \frac{N_{\text{decoy}}(t)}{N_{\text{target}}(t)}.$$

Afterwards, the FDR estimate is validated using human protein sequences as entrapment database. Target peptide identifications that are unmistakably ascribed to human proteins are deemed false positives and an entrapment false discovery estimate can be computed by: where $N_{\text{trap}}$ is the number of entrapment peptides among the target identification and $N_{\text{target}}$ is the number of all target identifications at scoring threshold *t*. R is the ratio of target database size over entrapment database size (number of peptides).


(5) $${\text{FDR}}_{\text{trap}}(t)=\frac{N_{\text{trap}}(t)}{N_{\text{target}}(t)}\cdot R,$$

Moreover, we append the post-processing software Percolator (version 3.05.0) (Käll *et al.* 2007) to investigate target decoy separation enhanced by support vector machines for Mistle and MSFragger. Parameters are used as suggested by the Fragpipe pipeline (with following flags set: --only-psms, --no-terminate, and --post-processing-tdc). FDR estimation is performed by Percolator. Features provided to Percolator include all the statistics collected by Mistle during the search. These range from various similarity and delta scores (see Section 2.2.1) to relevant metadata such as peptide length and precursor mass. The significance of individual features is discussed in the Section 3.4.

The impact of retention time features is evaluated separately with Percolator. Here, we use DeepLC (version 1.2.1) (Bouwmeester *et al.* 2021) to predict retention times, which are compared to the measured retention times of the experimental spectra. Absolute, squared and log distance, as well as a relative difference between measurement and prediction are calculated and added as columns to the input of Percolator.

### 2.4 Run time and memory consumption evaluation

All searches are performed on a Debian 5.10.113-1 system with an Intel(R) Xeon(R) Gold 5120 CPU @ 2.20 GHz and RAM of type DDR4, and with the data residing on an SSD. Eight threads were provided for each tool to make use of.

## 3 Results

### 3.1 Run time and memory performance

Time and hardware resources can become a critical factor when looking at large metaproteomes, covering thousands of species. For the moment, we evaluate run time and memory performances of all search software on a human consensus library and the two small lab-assembled microbiomes, 9MM and SIHUMIx. Feasibility for larger databases is discussed later on.

#### 3.1.1 Run time

MSFragger is currently one of the most popular and time-wise best performing database search algorithms. We use MSFragger as representative of sequence database search algorithms in contrast to the spectral library search algorithms we evaluate against. Note that spectral library search faces inherently different bottlenecks, e.g. data loading and continuous index construction, since it does not have the whole database information immediately, unless it loads every spectrum into RAM. At the same time, no sequence processing is required, e.g. protein digestion. As for the spectral library search engines, we compare Mistle to SpectraST, as it is a stable and popular option among spectral search software. Additionally, we benchmark msSLASH, which has been recently developed and introduces massive run time improvements by using Locality-Sensitive Hashing (Wang *et al.* 2020).

**Figure 3.**
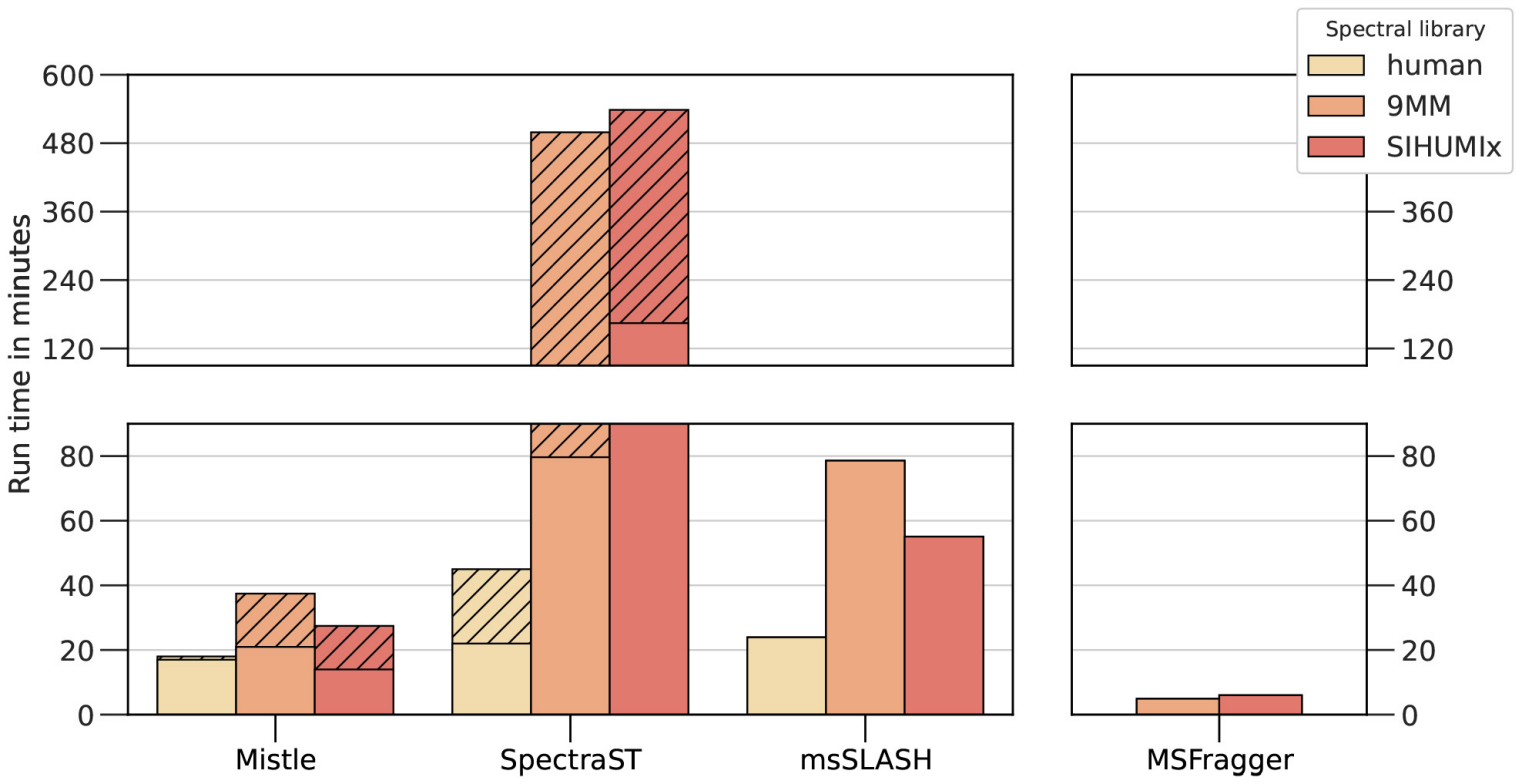
Total run time of all experimental search files measured for human, 9MM, and SIHUMIx libraries. Indexing time is indicated by stripes, whenever a separate index instance is constructed and saved to disk as intermediary step. Mistle performs the queries faster than any spectral library search engine (left side) but is slower than the database search algorithm MSFragger (right side).

Figure 3 compares the time required to search all experimental files between all four algorithms, split according to the spectral library. In all cases, Mistle is faster than the other spectral library search algorithms. The gain in performance is more significant the larger the library is. The metaproteome libraries are approximately 30 times larger (in terms of MS/MS spectra) than the human library, which is most notable in the increase in index construction time for Mistle and SpectraST. Mistle outperforms SpectraST by a factor of 2 (human library) to >10 (metaproteomes) and msSLASH by a factor of 2 when searching the predicted metaproteomes.

This places Mistle on a level comparable to the database search algorithm MSFragger, which is still a few times faster. Turning off MSFragger’s mass calibration and parameter optimization reduces its run time even further down to 1.9 min (less than half), highlighting that spectral search cannot quite match the speed of highly optimized database search. Reasons for that are: (i) the large index construction and data reading times, which in case of Mistle make up half of the total run time measured, requiring multiple I/O operations to load and save spectra and their fragments; (ii) a more cost-intensive spectral similarity calculation; and (iii) a current lack of optimization for multiple search files that require reloading Mistle’s index partitions between runs. Combining consecutive searches into a single query immediately speeds up the process. We test this by concatenating the four 9 MM search files into a single search file and analyse it with Mistle. While still querying the exact same experimental spectra, the search time reduces from 21 min down to 7 min, which is almost as fast as MSFragger. Also, note that the fragment index of Mistle needs to be constructed only once for each spectral library. Hence, when more files (or spectra) are searched, the run time reduces in relation to the other tools. Indexing time is indicated by the striped section in Fig. 3 for Mistle and SpectraST. Note that for predicted spectral libraries, the indexing time gets overshadowed by the construction and prediction process of the spectral data itself, which is much more time consuming. For instance, the 9MM target and decoy libraries alone take around 16 h for the prediction with Prosit (10 630 095 spectra).

Additionally, we investigated the time spent in distinct parts of the search loops, finding that *mistle-search* (lower bar in Fig. 3) uses more than 90% of its time for loading the spectral index and constructing data structures. Conversely, Mistle spends <10% of the time performing spectral queries, i.e. candidate search in the precursor index, fragment matching, and the final rescoring of top-ranked candidates. This demonstrates a current bottleneck at index loading operations that could benefit from further optimizations in the future, such as the use of memory mapping. Still, time-wise the fragment index search of Mistle introduces major improvements to spectral library search and brings it into a feasible reach compared to sequence database search.

#### 3.1.2 Memory

**Figure 4.**
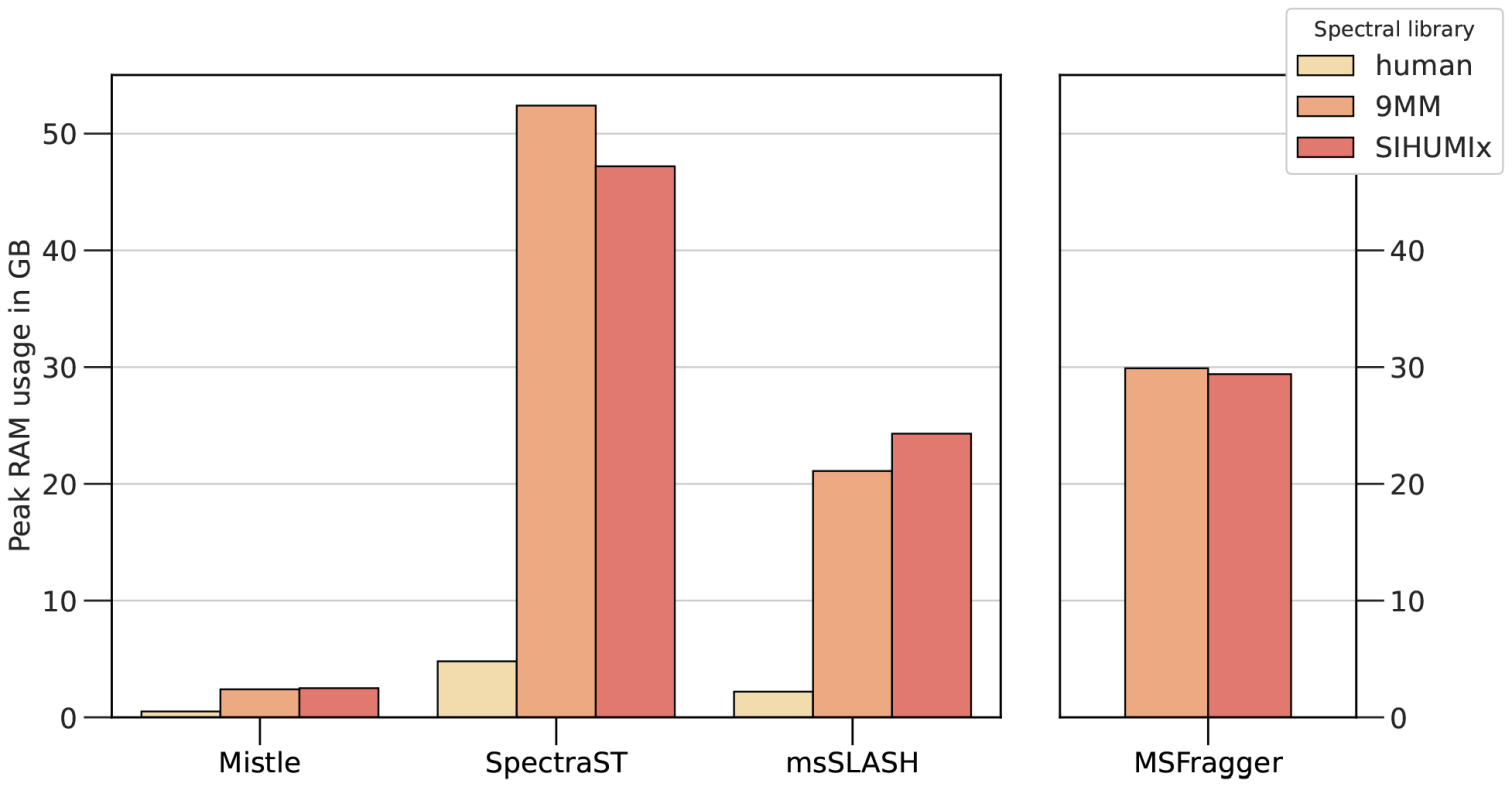
Memory consumption measured over all searches against the human, 9MM, and SIHUMIx libraries. Mistle performs much more RAM efficiently than all other tools with a 10 to 22-fold decrease in memory usage when compared to SpectraST and up to 10-fold decrease when compared to msSLASH, and >10-fold decrease when compared to MSFragger (run in default settings without memory restriction).

We analyse the memory requirements for all software, measured by the peak memory consumption across all runs against the human consensus library, 9MM and SIHUMIx, respectively. Metaproteome libraries are composed of the respective target spectra, the predicted human proteome (for entrapment) and the contaminants database with the predicted decoy libraries matching those. Figure 4 depicts the memory consumption in Gigabyte (GB). We find extraordinary memory improvements by the index partitioning and search scheduling method implemented in Mistle compared to all other search software, being around an order of magnitude more memory efficient. Compared to SpectraST, Mistle requires 10–22 times less RAM, performing the exact same task. Mistle effectively constructs a fragment index and performs searches in a 38 GB large MS/MS library (9MM) with less than 3 GB RAM, enabling queries on low performance computers, e.g. home laptops.

**Figure 5.**
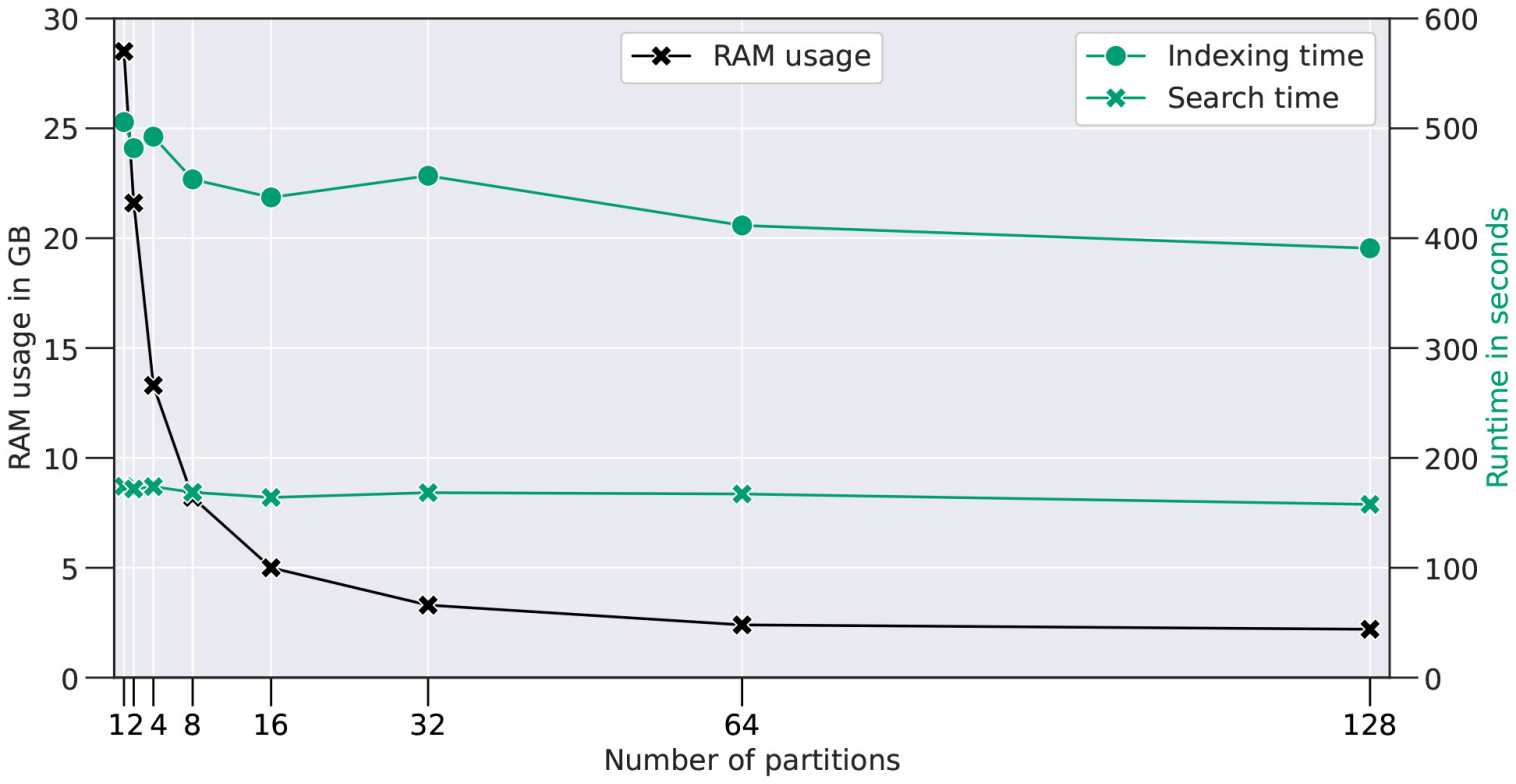
Memory consumption (black) and run time (green) shown for increasing numbers of partitions of Mistle’s fragment index for the 9MM target library. Peak RAM was measured, and the run time is divided into the time required to build the fragment index (dots) and search time (crosses) for the largest search file (9MM_Run_1.mgf).

We provide detailed information on how index partitioning and search scheduling affect run time and memory consumption in Fig. 5. While the run time remains relatively constant and even improves slightly with increasing partition count, the drop in memory consumption is eminent. The complete index (one partition) is comparable in size to MSFragger’s fragment index, though it holds additional information, such as peak intensity values. However, with the use of more than one partition, the RAM usage decreases according to the partition size. The usage converges to roughly 2 GB when using hundreds of partitions. This is the cached size of precursor index and query spectra together, while the fragment index size gets arbitrarily small by the partitioning. We use Mistle with 64 partitions in this study as it significantly reduces memory consumption with a stable run time, but the optimum is not reached with 64 partitions for this dataset.

Figure 5 also highlights the imbalance between search time and index construction time, as mentioned before. For a single search file, index construction takes almost three times longer than the spectral matching and ranking process, rendering Misle less efficient when the library is only queried once. On the other hand, many or particularly large MS/MS runs evaluated against the same metaproteome database benefit from an excellent search time on individual runs, where the fragment index (and spectral library) only needs to be constructed at the outset of an analysis.

RAM accessible to MSFragger can be restricted manually at an increase in run time. Restriction to 20 and 10 GB leads to a minor increase in search time of 10%–20%. Testing the limits, for 9MM we were able to reduce MSFragger’s memory consumption down to 3 GB (3.7 GB measured when running with FragPipe) before the program rejected the job due to insufficient memory. We measured a run time increase of around 65% (8.1 min in total). In the case of SIHUMIx, MSFragger crashes when setting the available memory to 3 GB. The lowest memory consumption achievable without an error was 4.6 GB. While these numbers are tolerable, large-scale metaproteomics reference databases are easily 100 times as large, such that memory requirements can have a profound impact on run time and feasibility. Mistle excels in memory-confined environments, where Mistle is not only much more performant than other spectral search software but also a real alternative to database search.

### 3.2 Quality control

Target decoy competition is the state-of-the-art method for quality assurance of PSMs, which we employ for all tested software. It is essential though, to verify that the FDR estimation is correct and remains stable across many datasets, especially when dealing with large search spaces. We put two mechanisms in place to validate the target decoy FDR. A yeast consensus library with annotated mass spectra serves as ground truth, which is queried against the inflated search space of 9MM (and entrapment) sequences. *Saccharomyces cerevisiae* is one of the species in the 9MM library. The second means of error estimation are entrapment sequences, which are concatenated to the target library. They provide an orthogonal FDR estimate (${\text{FDR}}_{\text{trap}}$) to confirm that the target decoy FDR remains stable across all performed searches. A mathematical description can be found in Section 2.3.3.

**Figure 6.**
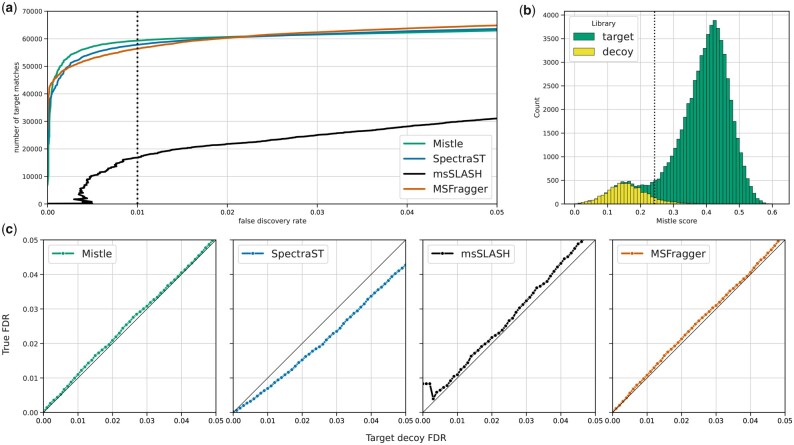
Statistics on yeast consensus spectra (ground truth) matched to the 9MM library (inflated search space): (a) PSM output numbers over FDR for all software; (b) score distribution of rank-1 target and decoy PSMs identified by Mistle; (c) true FDR (derived from peptide annotation provided for the yeast spectra) over target decoy FDR estimates. The black diagonal line displays where true FDR and target decoy estimate align perfectly. The vertical dotted lines in Figures (a) and (b) display the 1% FDR threshold.

Figure 6 shows the performance of all tools on the yeast ground truth dataset. We evaluate the number of PSMs at various target decoy FDR thresholds (Fig. 6a) and compare them with the true FDR (Fig. 6c) measured according to the peptide annotations in the consensus library. All algorithms except msSLASH identify high numbers of yeast spectra at 1% FDR, but Mistle identifies the most (82% of all search spectra). The true FDR is well reflected by the target decoy FDR estimate for all methods. Merely, in the case of SpectraST’s f-value the FDR is slightly overestimated, indicating that the cut-off is too stringent but otherwise correct, for this dataset. Even msSLASH, which struggles with peptide identification still maintains a relatively accurate FDR estimation. In conclusion, the spectral prediction approach for entire metaproteome sequence databases, including the MS/MS prediction of decoy sequences, provides the foundation for highly effective quality control. FDR estimation remains accurate no matter which search engine is used to perform the matching. Of course, the same holds for MSFragger when scoring target and decoy sequences.

In addition, we investigated the effect of different scores tracked by Mistle on the FDR. The PSM distribution of Mistle’s main scoring function (*average bias-adjusted similarity*, see Section 2.2.1) is depicted in Fig. 6b. The scores follow a bimodal distribution and decoy scores belong to one of the modes. This is another indication of a clean separation of true and false target PSMs by Mistle. Interestingly, we discovered that removing the *reflection score* from the *bias-adjusted similarity* improves PSM output, but at the same time slightly underestimates the true FDR. The dot product alone does not suffice to distinguish target and decoy matches properly in this enlarged search space. This is reflected by the poor performance of msSLASH, which uses a dot product of log-scaled peak intensities, and we can confirm this with Mistle’s dot product of square root transformed intensities. PSM output numbers for the standard *bias-adjusted similarity* and the dot product are shown in the Supplementary Material.

**Figure 7.**
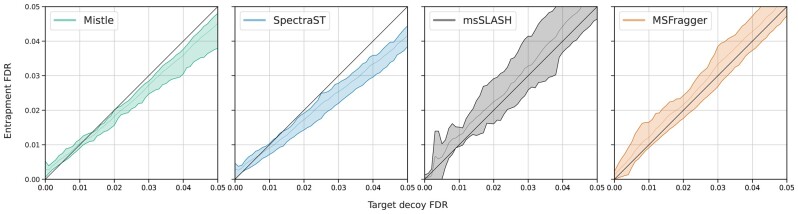
Entrapment FDR over target decoy FDR measured across all searches against the predicted metaproteome libraries (9MM and SIHUMIx). The range in entrapment FDR is displayed by the colored area for any target decoy FDR interval with the average displayed by the inner line. The black diagonal line (slope = 1) indicates the desired scenario, where the two FDR estimates align perfectly.

The entrapment sequences added to 9MM and SIHUMIx allow another measurement of false discoveries, as identified peptides that exclusively match to human proteins are most probably wrong. We ensured that the human entrapment spectra follow a similar distribution to both 9MM and SIHUMIx (regarding precursor mass ranges, charge types and number of peaks; shown in the Supplementary Material). Therefore, entrapment sequences fulfill the same role as decoy peptides, but are real biological peptides rather than computationally generated ones. Figure 7 shows the entrapment FDR over the target decoy FDR as a range (minimum, maximum and average) across all 9MM and SIHUMIx runs. For the most part, the entrapment FDR lies close to the target decoy FDR without much variation. The diagonal line indicates where the two FDR estimates are equal. msSLASH appears to have a higher variance in that regard, which we attribute to the smaller numbers of significant PSMs. SpectraST again, slightly overestimates the FDR rates with decoy sequences. Overall, the two orthogonal FDR estimates agree consistently, such that we expect little deviation from the true FDR throughout all the performed searches. Target decoy competition appears to be a well suited error estimation method for predicted spectral libraries of metaproteomes.

We conclude that Mistle’s scoring function descriminates well between true and false matches. Target scores are well separable by the bimodal distribution of their scores. If such behavior manifests throughout more and specifically larger datasets, omitting target decoy competition might be an eligible option. A mixture model approach as for example discussed by Nesvizhskii (2010) might realize FDR estimation equally well. Dropping decoy sequences altogether would reduce computational resources needed for library prediction, fragment index construction, and spectral search, enabling metaproteomic studies of much larger scale. Furthermore, FDR validation with the ground truth yeast spectra and entrapment sequences demonstrate the high quality of spectral matches with predicted peak intensities. Regardless of which search engine is used, a high quality of true peptide identification persists, which further support the proposed prediction workflow.

### 3.3 Peptide identification

**Figure 8.**
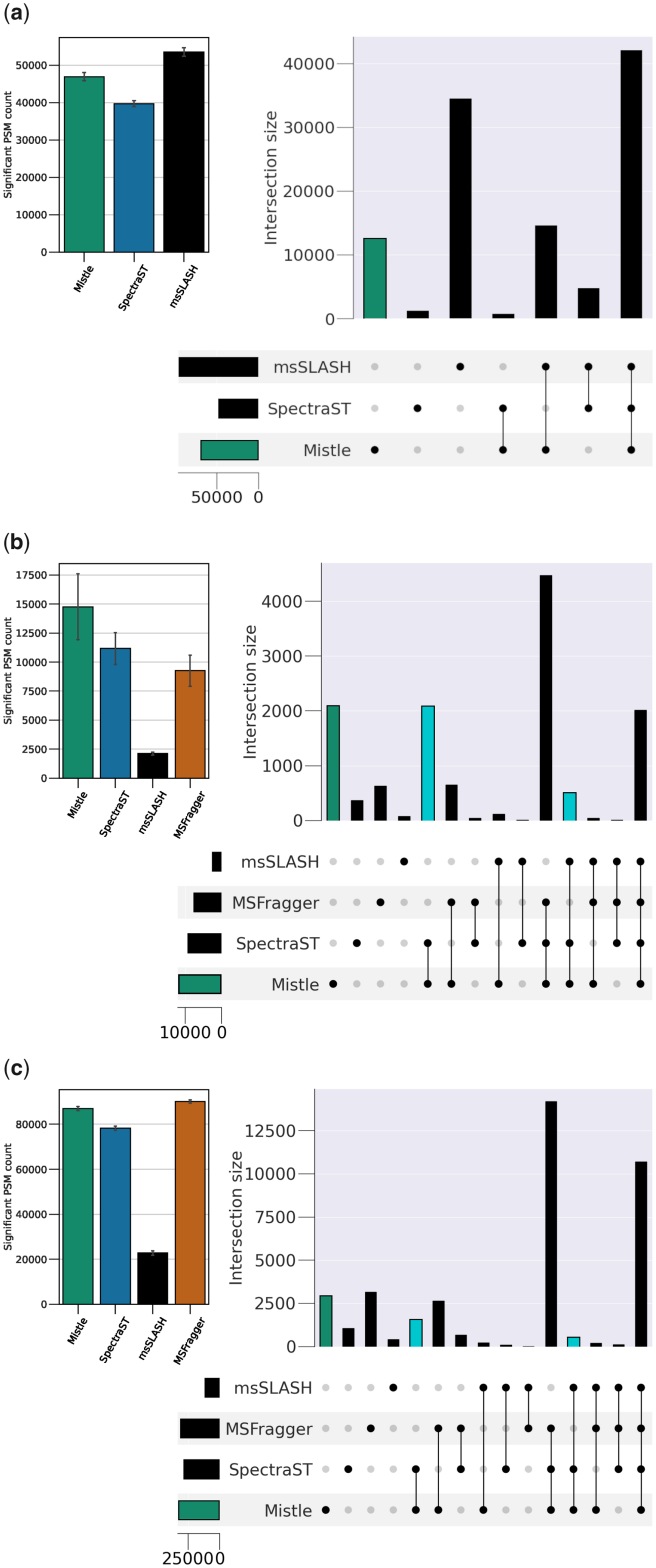
PSM and peptide output for the (a) human, (b) 9MM, and (c) SIHUMIx libraries. PSM counts are averaged across all search files based on the software-specific scoring thresholds at 1% FDR (without rescoring), shown on the left. Sets of identified peptides are depicted with an upset plot on the right (all searches combined). The individual bars show the set of peptides distinct to each software and the overlap between them. Peptides unique to Mistle are shown by the first bar. The overlap between the spectral search software (Mistle and SpectraST, as well as Mistle, SpectraST, and msSLASH) is highlighted.

We investigate peptide identification rates on PSM level and peptide level for all three datasets and all algorithms at 1% FDR. The PSM numbers are averaged across all search files queried with error-bars shown in Fig. 8 (on the left) for each dataset. On the right, the sets of distinct peptides and the overlap between the search algorithms are shown with an upset plot. In all cases, Mistle is extremely sensitive being the best or second best search engine in terms of PSM output. The identification rates range from 23% (9MM) to 73% (human). In comparison to the CAMPI benchmarking study, we observe fewer significant PSMs at 1% FDR: <100 000 for all search engines and files, whereas approximately 120 000 were identified in the CAMPI study. However, our library setup is more restrictive in terms of modification and permitted length of peptides—compare Section 2.3 with Van Den Bossche *et al.* (2021)—making it hard to judge the difference in raw numbers. Additionally, the statistics for S05 and S06 search files in the CAMPI study were obtained by database search with X! Tandem (Craig and Beavis 2004), which uses a two-stage search strategy, likely yielding additional hits.

When querying the predicted metaproteome libraries, Mistle identifies the largest sets of distinct peptides with SpectraST and MSFragger following closely behind. Only for the human spectral library msSLASH identifies more peptides than Mistle with its log-scaled dot product. However, the discriminating power of the dot product significantly drops when searching large search spaces as explained earlier. msSLASH identifies only a fraction of peptides querying the metaproteome libraries compared to all other tools. Scores accounting for the dot bias (Mistle and SpectraST) perform much better. Mistle consistently produces more significant hits than SpectraST, which we attribute to the slight FDR overestimation with the *f*-value. Qualitative differences between the two tools are discussed in Section 4. In general, Mistle and SpectraST show an elevated overlap when looking at their distinct peptide intersections, most notable in the 9MM study. The overlap between spectral search software (highlighted light blue in Fig. 8) indicates a striking number of peptides that is identified only through spectral search by matching peak intensities. Even though msSLASH identifies only a small set of peptides, its overlap with the results of Mistle and SpectraST is much more pronounced than its overlap with MSFragger. As a consequence, not only does Mistle identify more than 2000 peptides in each of the metaproteomes, which are completely unique to Mistle’s scoring function, but the elevated overlap between spectral search software also hinges at a non-negligible number of characteristic peptides that are different from the database search results. These findings validate the reliability and novelty of the spectral library prediction workflow, no matter which spectral search engine is used. An elaborate spectral similarity function is nonetheless indispensable.

**Figure 9.**
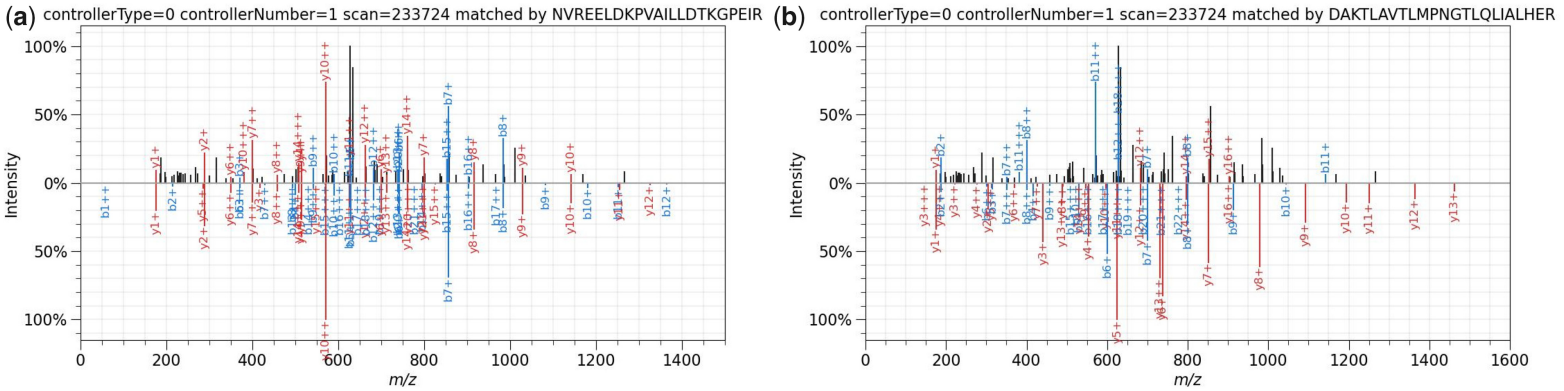
Mirror plot of a PSM identified by (a) Mistle and (b) MSFragger. In each case the top spectrum is the same experimental spectrum, scan 233724 of S05.mgf file and the bottom spectrum is the matched peptide spectrum from the SIHUMIx database with the peak intensities predicted by Prosit. Mistle finds a reasonable candidate with high spectral similarity of 0.52 and *reflection score* of 0.84. MSFragger identifies a decoy sequence with a hyperscore of 12.6. The spectral similarity to the predicted spectrum is very low with 0.09 and the *reflection score* is 0.29

When examining specific differences in PSMs, we uncovered some instances where spectral intensity-based matching produces a clearly better match than database search using MSFragger. Figure 9a shows such an example from the SIHUMIx query (top of the mirror plot) paired with the spectral prediction from Prosit (bottom) of the peptide match found by Mistle, and Fig. 9b shows the same spectrum matched by MSFragger. The near perfect overlap between b and y ion intensities (*reflection score* of 0.84) suggests that Mistle identified the correct peptide. SpectraST corroborates Mistle’s finding. In contrast, the PSM suggested by MSFragger for the same query spectrum is an apparent mismatch to a decoy peptide. This is only obvious given the peak intensity predictions by Prosit, as the theoretical b and y ions cover only a fraction of all peaks in both cases. Note that post-processing tools like Percolator eliminate this false discovery, but cannot recover the correct match.

### 3.4 Post-processing

Percolator is a renowned post-processing software, which trains a linear support vector machine to separate target and decoy matches (Käll *et al.* 2007). By doing so, Percolator optimizes the target PSM output through several iterations of semi-supervised learning with cross-validation. We append Percolator to Mistle’s and MSFragger’s output, provided in pin-tab format that lists the search scores and statistics as features for the classifier.

This leads to an increase in Mistle’s target output by 4.4%–17.4% compared to a threshold on the *average bias-adjusted similarity*. Smaller impact is seen for the predicted metaproteome libraries, where Percolator boosts the performance on average by 8.8% (9MM) and 6.4% (SIHUMIx). For the human spectral library, the PSM output was significantly increased with an average of 15.3%. MSFragger yields slightly more PSMs (4%–8%) than Mistle, almost doubling its output on the larger 9MM queries compared to a hyperscore cut-off. A direct comparison of PSMs identified with and without Percolator is provided in the Supplementary Material.

In terms of unique peptide identifications, both tools have very comparable results after rescoring. Mistle even identifies more distinct peptides in the SIHUMIx queries than MSFragger, despite having fewer PSMs. About 10% of peptides are specific to each search engine and remain undetected by the other. This once more highlights the merit of exploring orthogonal search approaches as standard database search alone is not fully comprehensive. The proposed spectral library workflow is sensitive to a different set of peptides, which may well be of biological significance. Corresponding figures are provided in the Supplementary Material.

Percolator also returns its feature weights across all its cross-validation splits, which can be related to the feature importance. Note, however, that some features are highly correlated, making it difficult to judge their exact contribution. Still, we can confidently identify the following trends: Direct spectral similarity measures have a great influence with the *reflection score* being more important in case of the predicted libraries. This is coherent with the library setup, as only b and y ions are predicted, and missing peaks do not influence the *reflection score*. Respective score biases, e.g. dot bias, have consistently negative weights as is expected, favoring PSMs with high similarities and low biases. Occasionally, statistics like the shared peak count and *m*/*z* standard deviation of matched fragments had a contribution to target decoy separation. Smaller importance was given to delta scores, hyperscores, and mass differences on precursor level. For queries against the human spectral library, the dot product was the most important feature by far. This coincides with the good performance of msSLASH, which uses a dot product with log-scaled intensities. Interestingly, dot product seems to perform significantly better than the similarity on this low-resolution library. Remember that the similarity uses an *m*/*z* standard deviation rather than a fixed *m*/*z* tolerance and still penalizes mass difference for matched fragments. Moreover, biases have lower impacts, as both query and reference spectra contain all types of ions and noise peaks. This demonstrates that Percolator is a great addition to Mistle for cases when the *average bias-adjusted similarity* generates a slightly smaller target output.

In addition, we investigate the effect of retention time features added to Percolator. We use DeepLC for retention time prediction, as described in Section 2.3.3, for peptide matches from SIHUMIx and 9MM. Note that while Prosit can predict retention times, they are currently not part of the spectral libraries. Both search engines receive a moderate but consistent boost of 1%–3% to their PSM output when retention time features are present. Percolator emphasizes that the relative retention time difference is one of the most important features for classification. At the moment, this is an untapped advantage that spectral libraries have over database search. Including retention time information in the library and calculating the corresponding features directly during the search eliminates the need for tedious and manual prediction of features post-search. This represents a potential future improvement to the current workflow.

With rescoring, Mistle now outperforms msSLASH in terms of PSM output for the human library in most cases (15 out of 18 search files). Mistle is the only spectral search engine in this study that supports the Percolator-readable pin-tab format. This makes Mistle extremely versatile, likely performing well on any new dataset and also when search parameters are chosen inadequately.

## 4 Discussion

Coping with complete predicted libraries for metaproteomics, covering more than 10 million peptide MS/MS spectra, proves to be a challenging task for spectral search software. Especially, analyzing the large candidate space in RAM is very demanding and might just fail when looking at more diverse microbiomes. We provide proof of concept that our approach works well for two mock-communities, turning all peptides from their sequence database into MS/MS spectra. Despite the large datasets, the presented search algorithm, Mistle, is extremely memory efficient due to an effective index partitioning technique. The memory requirements of Mistle are an order of magnitude smaller than those of all other spectral search software. In terms of run time, Mistle is up to 10 times faster than SpectraST, up to 2 times faster than msSLASH, and stays close to the ultra-fast database search algorithm MSFragger. Even though Mistle cannot quite match the run time of MSFragger, the challenges faced by each approach are quite different, and the improvements introduced by our algorithm are nonetheless significant.

Investigating peptide identification, we find cohesive results between Mistle and SpectraST identifying high numbers of PSMs, but Mistle finds consistently more unique peptides (see Fig. 8). The elevated peptide overlap between SpectraST and Mistle, and to some degree even msSLASH, reinforces the idea of spectral matching being able to identify peptides that standard database search cannot. Delving into this, we presented an example spectrum visibly attaining a much more reasonable match using our approach, when compared to MSFragger, which identifies a decoy peptide (see Fig. 9).

We ensured a high quality of spectral matches by verifying the target decoy FDR with annotated yeast spectra. The FDR is estimated accurately across all algorithms, legitimizing the use of spectral predictions (target and decoy sequences) when confronted with large search spaces. Mistle’s *average bias-adjusted similarity* (see Section 2.2.1) produces excellent bimodal distributions of PSM, which enable a clean separation of true and false discoveries, while at the same time being highly sensitive and accurate. Entrapment sequences spiked into the target library confirm the soundness of the target decoy approach for FDR estimation throughout the numerous searches. Although we put our focus on evaluating tool-specific scoring functions, Mistle can also be coupled to Percolator to boost identification rates even further, reaching up to 17.4% additional hits. This is another advantage of Mistle compared to other spectral search software. Still, there is room for further refinement of features, e.g. by integrating retention time predictions in the reference libraries. Our tests suggest that adding retention time features increases PSM performance again by up to 3%. In conclusion, we prove the applicability of Mistle to common lab-assembled studies and have reason to believe that the workflow will perform well for even larger metaproteomics studies.

Currently, the main shortcoming resides in building the spectral library as an in-between step, which is resource intensive (time, and disk space). Additionally, loading times from disk takes more than 90% of the total search time, which is the reason for slightly increased run time compared to state-of-the-art database search methods like MSFragger. A way to mitigate long loading times is to distribute search tasks among several servers, each permanently keeping an index partition in RAM. As a positive side effect partitions can then be queried in parallel without any I/O operations.

The prediction workflow produces satisfactory results no matter which search engine is used, as long as the spectral similarity measurement is carefully selected and tested. However, Mistle has an excellent trade-off between run time and memory consumption and outperforms SpectraST in that aspect by far. Mistle is best used for repeated scans on the same metaproteomic environment, like for instance SIHUMIx, such that the spectral library and search index are constructed only once. The sequence database and parameters can be chosen generously to be very comprehensive, and the performant search algorithm excels at multiple MS/MS runs against the same library. Spectral library construction using Prosit can be optimized further, e.g. by calibrating the collision energy based on the experimental raw data. The low memory consumption makes Mistle feasible for studies on low performance machines, e.g. laptop computers, but also allows much larger protein databases to be analysed, where the competing tools are quickly overchallenged. Of course, Mistle, being a spectral search engine at heart, can be used on any experimental spectral library, too. We highlight this by using Mistle to query 18 experimental MS/MS files to the NIST human consensus library. Although this represents only a small spectral library, the boost in performance and low memory footprint suggest that Mistle is well suited for far more comprehensive libraries.

There are small qualitative differences between SpectraST and Mistle, which arise from different pre-processing steps and the scoring functions used, e.g. neighboring bin matching for SpectraST and peak matching using a Gaussian distribution for Mistle. Turning off most pre-processing features and using the native dot product for scoring produces nearly identical results between SpectraST and Mistle. We also suspect that the current search setup with a separate target and decoy library might affect the discriminating power of SpectraST’s f-value. Thereby, differences in dot products [ΔD, see Lam *et al.* (2007)] are not calculated between target and decoy matches and may be a reason for the slight reduction in sensitivity. At this point, we conclude that the differences in PSM scoring play a minor role when looking at the overall identification of unique peptides in the samples. Figure 8 demonstrates a large overlap in the findings of both spectral library search engines. Depending on the dataset, Mistle agrees with the peptides found by SpectraST in 88%–95% of the cases.

With our tool, we open the door to investigate much larger metaproteomes, e.g. the human gut microbiome, with the help of predicted spectral libraries. In an ideal setting, spectral library prediction is set up to cover the entire metaproteome comprehensively with carefully selected parameters in accordance with the wet lab. Then, the effect of treatments, different samples or patient groups can be perpetually analysed by spectral search with Mistle producing reliable peptide identification in the large search space at fast rate, and without being memory intensive.

## Supplementary Material

btad376_Supplementary_Data
