# Supplementary material for: Mistle: bringing spectral library predictions to metaproteomics with an efficient search index
Source: Bioinformatics. 2023 Jun 9;39(6):btad376. doi: 10.1093/bioinformatics/btad376 (PMC10313348; doi:10.1093/bioinformatics/btad376)
Supplement: btad376_Supplementary_Data [file btad376_supplementary_data.pdf]

Supplementary Material

Mistle: bringing spectral library predictions to metaproteomics with an efficient search index

Yannek Nowatzky [1], Philipp Benner [1], Knut Reinert [2, 3] and Thilo Muth [1,*]

August 2022

[1]eScience S.3, Bundesanstalt für Materialforschung und -prüfung, Unter den Eichen 87, 12205 Berlin, Germany and
[2]Department of Mathematics and Computer Science, Free University Berlin, Takusstraße 9, 14195 Berlin, Germany and
[3]Max Planck Institute for Molecular Genetics, Ihnestraße 63-73, 14195 Berlin, Germany.

Contents

1 Summary **4**

2 Methods **4**

2.1 Continuous index construction 4
2.2 Search Loop 4
2.3 Datasets 6
2.3.1 9MM 6
2.3.2 SIHUMIx 7

3 Supplementary Figures **8**

List of Figures

1 Schematic depiction of the fragment index partitioning into 4 sub-indices based on precursor index ranking. All peaks from a parent entry (highlighted on top) are listed inside the fragment sub-index (partition) determined by the parent's m/z. For search and construction, only a single partition and the precursor index need to be loaded into RAM. 8

2 Visualization of AVX2 fused multiply-add operation performed vertically on 8 intensity values (32-bit floats). The process associated with data loading into and retrieving from 256-bit destinations is displayed for a single fragment bin. *Gather* and *scatter* instructions are only available for AVX512 compatible CPUs. This computation replaces step 4 of the inner search loop described in 2.2. 8

3 Statistics for the predicted 9MM spectral library. Number of spectra, distributions of precursor m/z and peak counts are shown individually for all charge types (charges 2 to 4). 9

4 Statistics for the predicted SIHUMIx spectral library. Number of spectra, distributions of precursor m/z and peak counts are shown individually for all charge types (charges 2 to 4). 9

5 Statistics for the predicted human spectral library. Number of spectra, distributions of precursor m/z and peak counts are shown individually for all charge types (charges 2 to 4). 10

6 Target PSM output over FDR for scores tracked by Mistle: *Average bias-adjusted similarity*, *bias-adjusted similarity* and dot product. The dot product is insufficient in separating target and decoy matches at a high sensitivity. Scores accounting for the dot bias perform much better. For more details about the scores refer to the main article. 11
7 PSM output at 1% FDR with and without rescoring for the 9MM dataset. Without rescoring the cut-off is based on the *average bias-adjusted similarity* in case of Mistle and the *hyperscore* in case of MSFragger. Rescoring PSMs with Percolator leads to an average increase of 8.8% in hits for Mistle and almost doubles MSFraggers PSM output. Retention time (RT) features added to the PSMs lead to another small increase of 1% to 3% in both cases. 12
8 PSM output at 1% FDR with and without rescoring for the SIHUMIx dataset. Without rescoring the cut-off is based on the *average bias-adjusted similarity* in case of Mistle and the *hyperscore* in case of MSFragger. Rescoring PSMs with Percolator leads to an average increase of 6.4% in hits for Mistle and 9.4% for MSFragger. Retention time (RT) features added to the PSMs lead to another small increase of 1% to 3% in both cases. 13
9 Unique peptides identified by Mistle and MSFragger after rescoring the search results with Percolator. Both search engines identify comparable numbers of peptides, with MSFragger finding slightly more distinct peptides for 9MM queries (a) and Mistle finding more peptides for SIHUMIx queries (b). About 10% of peptides are specific to each search engine and remain undetected by the other. 14

1 Summary

This is the supplementary material to the article *Mistle: bringing spectral library predictions to metaproteomics with an efficient search index*. Additional explanation of the methods and datasets are provided. Moreover, supplementary figures supporting the study are attached in section 3.

2 Methods

In this section we provide additional information to some of method deployed in the main article, and elaborate the data processing steps. This serves as ancillary information, and is only fully comprehensible in combination with the main text.

2.1 Continuous index construction

As explained in the main article, the fragment index needs to be constructed continuously during the reading process of the spectral library.

This goes as follows: Each library spectrum is read one after the other, precursor information is added to the precursor index and all their peaks are streamed as fragment triplets to the corresponding index partition in binary format on disk. Missing (zero-intensity) theoretical fragment ions are not explicitly calculated from the peptide sequence and remain untracked unless they are provided within the spectral library.

The process is parallelized by having a single thread dedicated to reading while the rest are occupied by formatting and writing tasks. At this point, fragments are unsorted and not binned, but assigned to their partition. After the library has been read entirely, the precursor index is sorted by precursor charge and m/z, and the ID-to-rank mapping is established by a linear scan over the precursor list. Then, every partition is loaded again, one at a time, and fragments are sorted based on parent ranks, accessible via the ID. Afterwards, the partition is saved to disk in binary format. Binning only happens during the search, based on the user-specific fragment tolerance.

2.2 Search Loop

Let Q be a query spectrum with precursor m/z mz_Q and a set of peaks P_Q. We identify the best PSMs by computing the dot product to the reference library spec-

tra, carrying out the following steps:

(1) First, the range of library candidate spectra that lie within the precursor mass tolerance t is computed using the precursor index. A binary search on the sorted precursor entries swiftly finds the lower bound L and upper bound U, which is the rank of the first and last library spectrum with a precursor m/z $\in [mz_Q - t, mz_Q + t]$.

(2) Next, a scoring vector (scores) of length $U - L$ is allocated and initialized with all zeros. This way, the score of each candidate spectrum can be accessed from its rank R by subtracting L.

(3) Every peak $p = (mz_p, I_p) \in P_Q$ is searched in the fragment index by first determining the fragment bin matching its ion mass mz_p. Recall that inside each bin the fragments are stored in order of their parent ranks. A binary search then identifies the first fragment $f = (mz_f, I_f, \mathrm{ID}_{\mathrm{parent}}(\mathrm{f}))$ with a parent rank R greater than or equal to L (rank of the first candidate). R is derived from $\mathrm{ID}_{\mathrm{parent}}(\mathrm{f})$ via the ID-to-rank mapping.

(4) The fragment intensity I_f is multiplied with the query peak intensity I_p and the product is added to the score of the fragment's parent:

$$\mathrm{scores}[R - L] \leftarrow \mathrm{scores}[R - L] + I_p I_f.$$

The process is repeated with the next fragment in the bin until a fragment with parent rank greater than U is reached. This marks the end of candidates for that fragment bin.

(5) At the end, the scores equal the dot products between search spectrum Q and every candidate library spectrum covered by the partition. We rescore the best-scoring library spectra with an elaborate scoring function (see main article). Then, the X highest scoring library spectra are selected, and the corresponding peptides are returned as PSMs to Q. X, the number of output PSMs per query spectrum (X>0), is a parameter defined by the user.

The search function is parallelized matching each query spectrum on a separate thread. After all scheduled queries are performed, the resulting PSMs from all the partitions are concatenated and sorted by query ID. Matches assigned to the same experimental spectrum cluster together, and again only the top X ranked matches are retained, if multiple partitions produced hits for the same query.

SIMD intrinsics

Single instruction multiple data (SIMD) is a type of parallel computation, which simultaneously performs an operation on packed groups of data, instead of on every single data point individually (Amiri and Shahbahrami, 2020). Since their introduction to general-purpose processors, first by Intel's MultiMedia eXtensions (MMX) in 1996, it has been widely established as significant means to speed up performance (Amiri and Shahbahrami, 2020; Hassaballah *et al.*, 2008; Zhou and Ross, 2002).

The search algorithm requires many successive multiplication and addition operations when updating parent scores with peak intensity products. Consequently, SIMD extensions are an eligible option to improve our run time. We use the Advanced Vector Extensions AVX2 and AVX512 architectures, which support the fused multiply-add arithmetic operation (for 256-bits in C++: *_mm256_fmadd_ps*) for floating-point vectors, thereby updating multiple parent scores in parallel. A schematic version of the workflow for a 256-bit register is depicted in Supplementary Figure 2.

Explicitly, this is done by broadcasting a single 32-bit float, the query peak intensity value, into the 256 or 512-bit register (for 256-bits in C++: *_mm256_set1_ps*) and loading respectively 8 or 16 fragment intensities from the fragment-ion bin (using *_mm256_loadu_ps*). Then, the corresponding score values are inserted into another register. Finally, the multiply-add operation (using *_mm256_fmadd_ps*) is performed vertically on all 8 or 16 values at the same time: multiplying the query intensity with the individual fragment intensities and adding the result to the corresponding scores, which are extracted afterwards. The AVX512 instruction set provides a *gather* instruction to quickly access the values from the scoring vector and a *scatter* instruction to put them back in place after the computation. Note that the fragment bins need to be adjusted in their format to enable swift loading of intensity values.

2.3 Datasets

2.3.1 9MM

In 2013, Tanca *et al.* investigated the effect of sequence databases used to query shotgun proteomic results for diverse microbial communities. They evaluate their findings on a lab-assembled mock community of nine bacterial and eukaryotic species: *Escherichia coli, Pasteurella multocida, Brevibacillus laterosporus, Lac-*

tobacillus acidophilus, Lactobacillus casei, Enterococcus faecalis, Pediococcus pentosaceus, Rhodotorula glutinis, and . The 9MM dataset has since been used to evaluate metaproteomic pipelines. We utilize the sequence database (9MM_DB.fasta) and 4 search files (9MM_FASP.raw, 9MM_PPID.raw, 9MM_Run_1.raw, 9MM_Run_2.raw) provided in the original and the follow-up study, which can be found at http://www.peptideatlas.org/PASS/[illegible] and
http://www.peptideatlas.org/PASS/PASS00355 (Tanca *et al.*, 2013, 2014). Raw files were convered using ms-convert from the ProteoWizard software (Chambers *et al.*, 2012) with peak picking retaining the top 150 most intense peaks.

2.3.2 SIHUMIx

The extended simplified human microbiota (SIHUMIx), established by Krause *et al.* (2020), is a model community of eight species from the human intestine that account for most of the typical metabolic activities in the human gut. The model allows consistent and reproducible in-vitro cultativation, making it ideal to investigate the effect of treatments to the microbiome (Schäpe *et al.*, 2020). SIHUMIx consists of the species: *Anaerostipes caccae, Bifidobacterium longum, Bacteroides thetaiotaomicron, Blautia producta, Clostridium butyricum, Clostridium ramosum, Escherichia coli* and *Lactobacillus plantarum*.

As reference sequence database we use the one provided by the CAMPI challenge (Van Den Bossche *et al.*, 2021), which already includes decoy and contaminant sequences (Pride Project ID: PXD023217). Searches are performed on two large search files (S05, S06) from the CAMPI study, which yielded the most identified PSMs without fractionation.

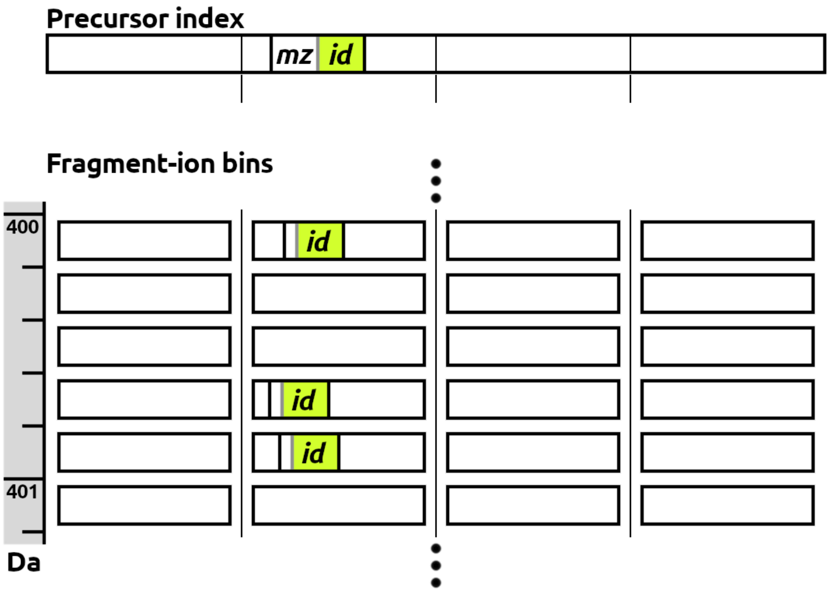

Figure 1: Schematic depiction of the fragment index partitioning into 4 sub-indices based on precursor index ranking. All peaks from a parent entry (highlighted on top) are listed inside the fragment sub-index (partition) determined by the parent's m/z. For search and construction, only a single partition and the precursor index need to be loaded into RAM.

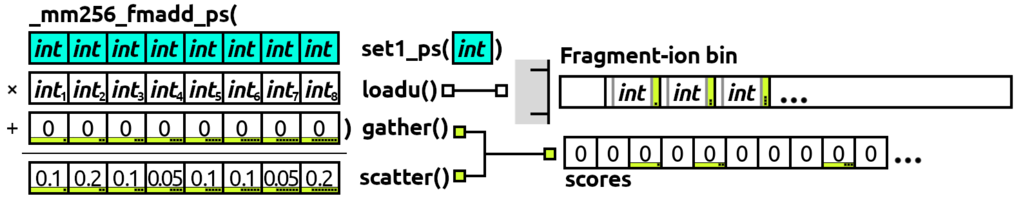

Figure 2: Visualization of AVX2 fused multiply-add operation performed vertically on 8 intensity values (32-bit floats). The process associated with data loading into and retrieving from 256-bit destinations is displayed for a single fragment bin. *Gather* and *scatter* instructions are only available for AVX512 compatible CPUs. This computation replaces step 4 of the inner search loop described in 2.2.

3 Supplementary Figures

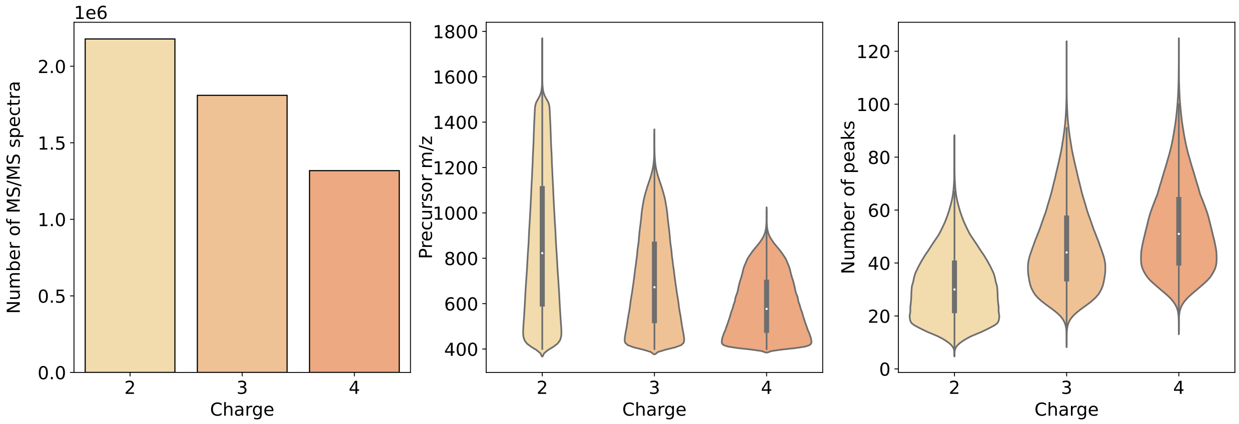

Figure 3: Statistics for the predicted 9MM spectral library. Number of spectra, distributions of precursor m/z and peak counts are shown individually for all charge types (charges 2 to 4).

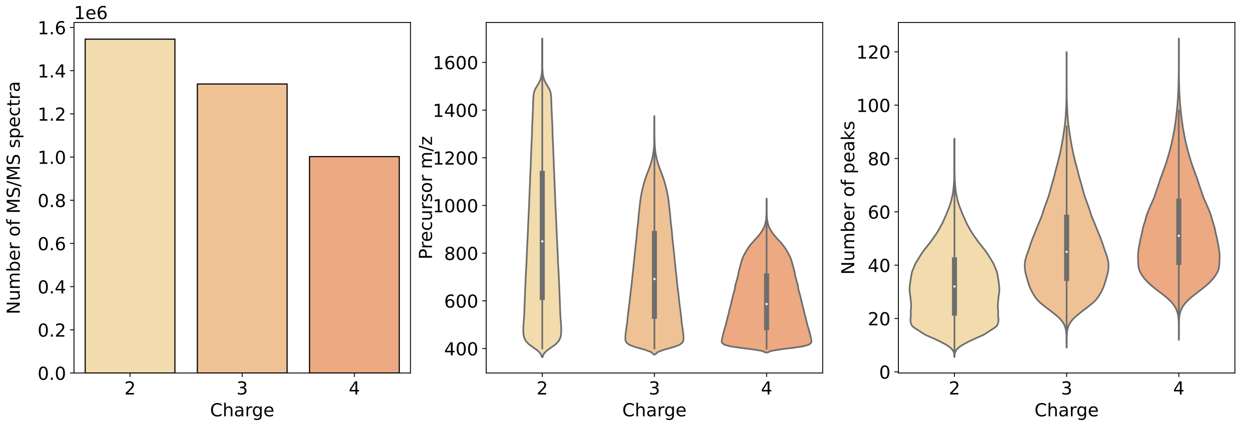

Figure 4: Statistics for the predicted SIHUMIx spectral library. Number of spectra, distributions of precursor m/z and peak counts are shown individually for all charge types (charges 2 to 4).

Peptide sets

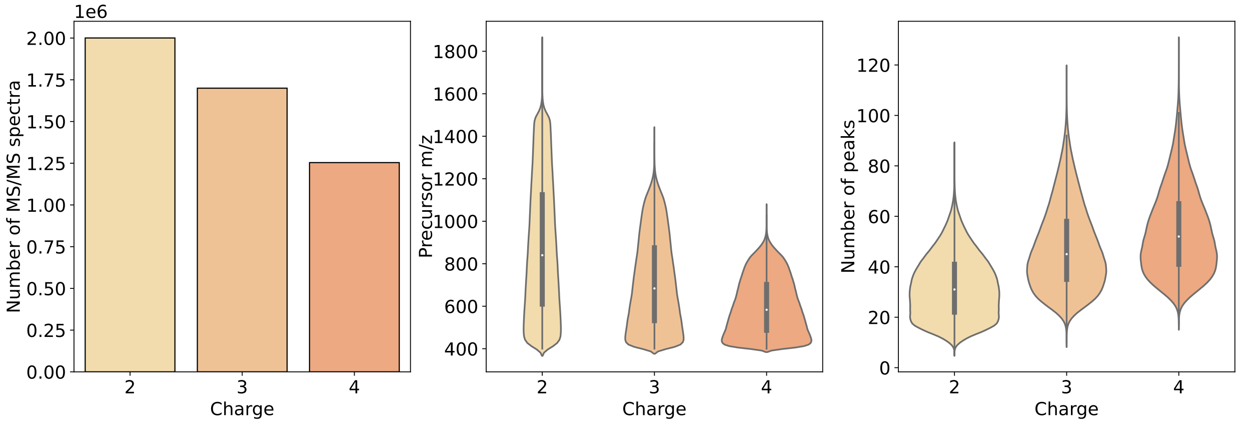

Figure 5: Statistics for the predicted human spectral library. Number of spectra, distributions of precursor m/z and peak counts are shown individually for all charge types (charges 2 to 4).

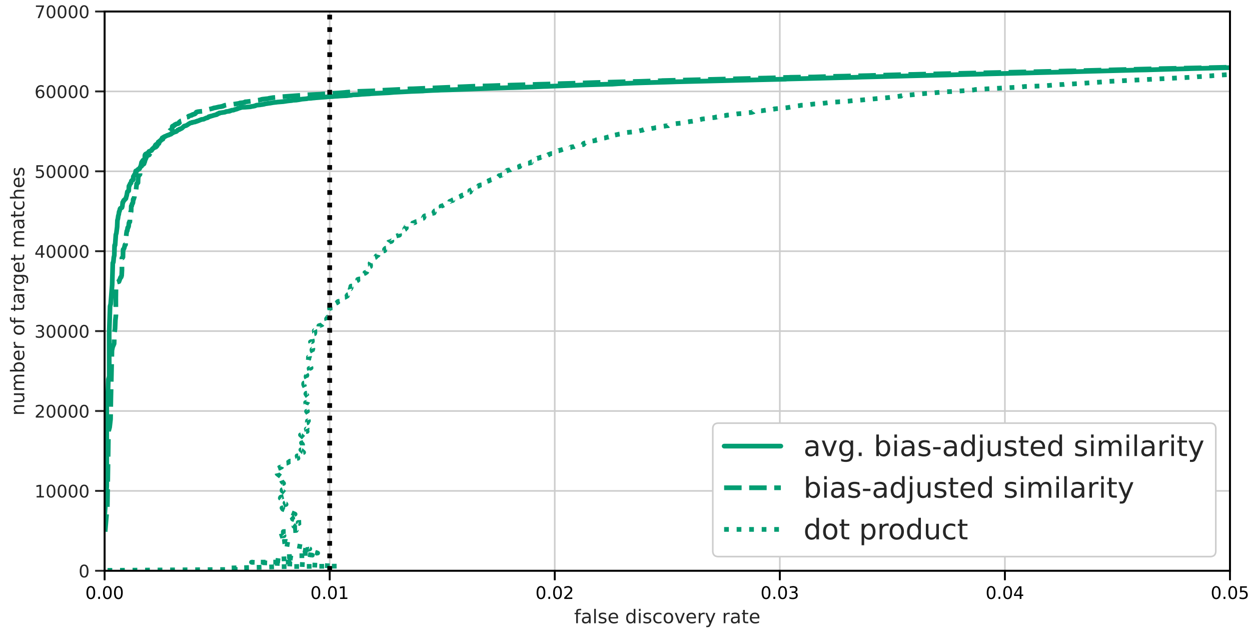

Figure 6: Target PSM output over FDR for scores tracked by Mistle: *Average bias-adjusted similarity*, *bias-adjusted similarity* and dot product. The dot product is insufficient in separating target and decoy matches at a high sensitivity. Scores accounting for the dot bias perform much better. For more details about the scores refer to the main article.

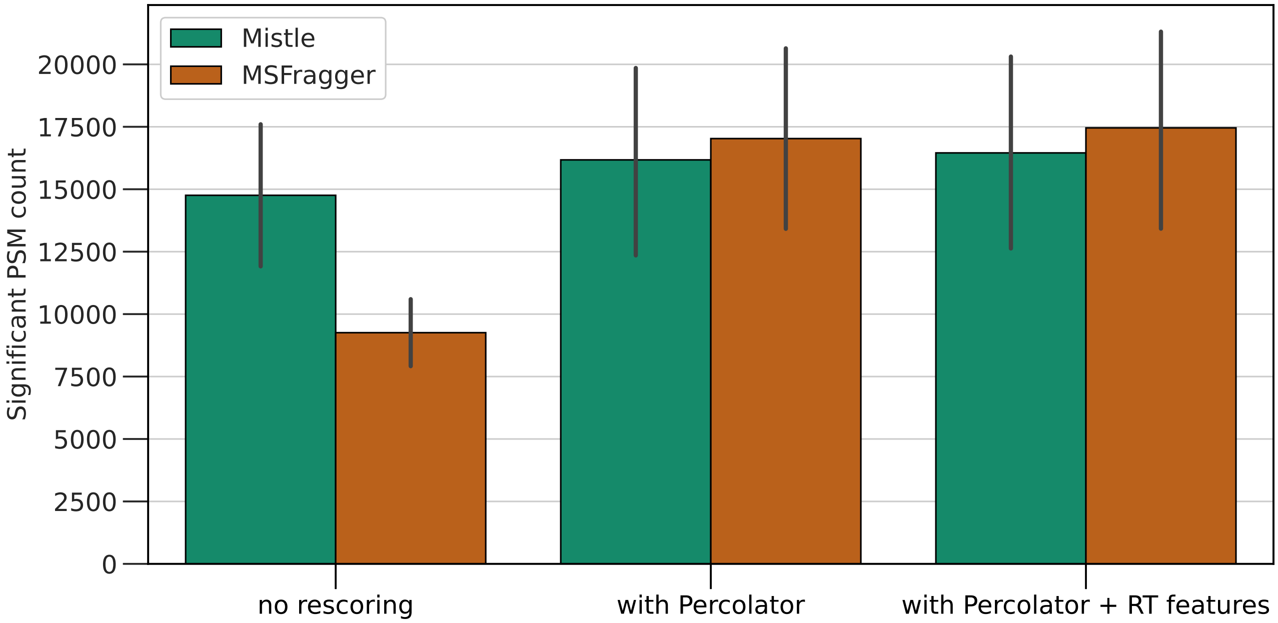

Figure 7: PSM output at 1% FDR with and without rescoring for the 9MM dataset. Without rescoring the cut-off is based on the *average bias-adjusted similarity* in case of Mistle and the *hyperscore* in case of MSFragger. Rescoring PSMs with Percolator leads to an average increase of 8.8% in hits for Mistle and almost doubles MSFraggers PSM output. Retention time (RT) features added to the PSMs lead to another small increase of 1% to 3% in both cases.

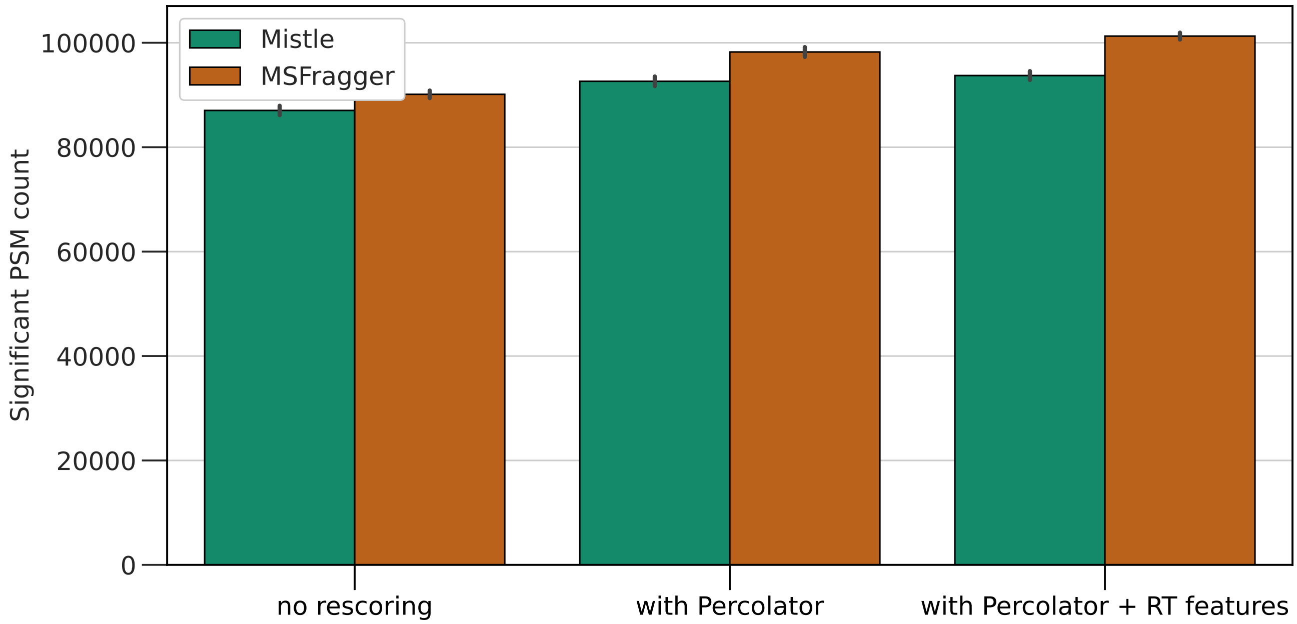

Figure 8: PSM output at 1% FDR with and without rescoring for the SIHUMIx dataset. Without rescoring the cut-off is based on the *average bias-adjusted similarity* in case of Mistle and the *hyperscore* in case of MSFragger. Rescoring PSMs with Percolator leads to an average increase of 6.4% in hits for Mistle and 9.4% for MSFragger. Retention time (RT) features added to the PSMs lead to another small increase of 1% to 3% in both cases.

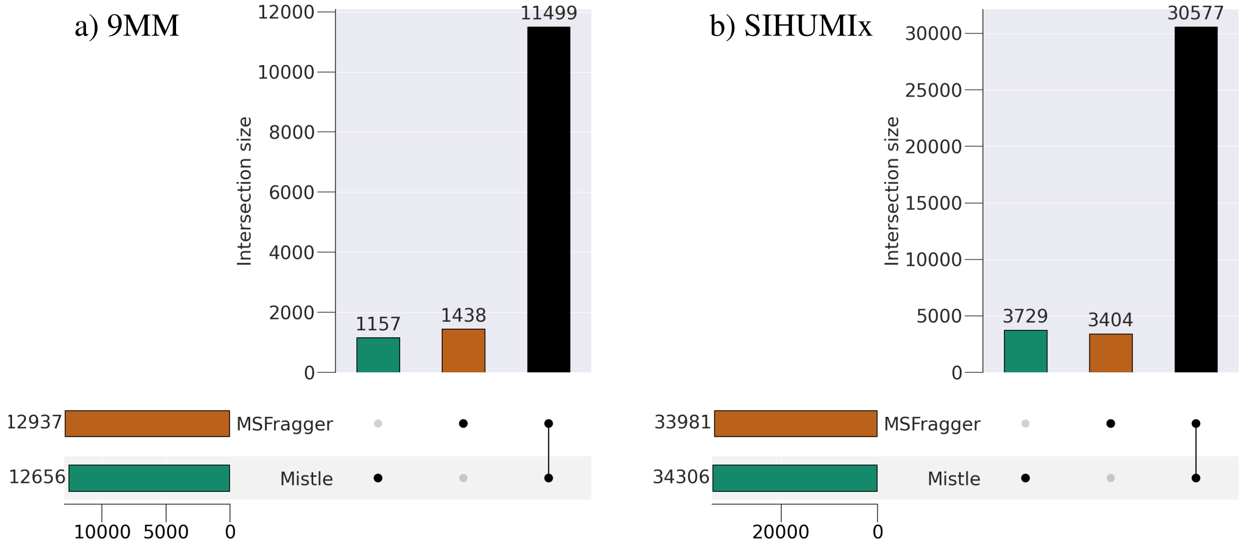

Figure 9: Unique peptides identified by Mistle and MSFragger after rescoring the search results with Percolator. Both search engines identify comparable numbers of peptides, with MSFragger finding slightly more distinct peptides for 9MM queries (a) and Mistle finding more peptides for SIHUMIx queries (b). About 10% of peptides are specific to each search engine and remain undetected by the other.

References

Amiri, H. and Shahbahrami, A. (2020). Simd programming using intel vector extensions. *Journal of Parallel and Distributed Computing*, **135**, 83–100.

Chambers, M. C. *et al.* (2012). A cross-platform toolkit for mass spectrometry and proteomics. *Nature biotechnology*, **30**(10), 918–920.

Hassaballah, M. *et al.* (2008). A review of simd multimedia extensions and their usage in scientific and engineering applications. *The Computer Journal*, **51**(6), 630–649.

Krause, J. L. *et al.* (2020). Following the community development of sihumix–a new intestinal in vitro model for bioreactor use. *Gut Microbes*, **11**(4), 1116–1129.

Schäpe, S. S. *et al.* (2020). Environmentally relevant concentration of bisphenol s shows slight effects on sihumix. *Microorganisms*, **8**(9), 1436.

Tanca, A. *et al.* (2013). Evaluating the impact of different sequence databases on metaproteome analysis: insights from a lab-assembled microbial mixture. *PloS one*, **8**(12), e82981.

Tanca, A. *et al.* (2014). A straightforward and efficient analytical pipeline for metaproteome characterization. *Microbiome*, **2**(1), 1–16.

Van Den Bossche, T. *et al.* (2021). Critical assessment of metaproteome investigation (campi): a multi-laboratory comparison of established workflows. *Nature communications*, **12**(1), 1–15.

Zhou, J. and Ross, K. A. (2002). Implementing database operations using simd instructions. In *Proceedings of the 2002 ACM SIGMOD international conference on Management of data*, pages 145–156.
